# Viral suppression among adolescents and adults living with human immunodeficiency virus in Ethiopia during the test-and-treat era

**DOI:** 10.1371/journal.pone.0362375

**Published:** 2026-11-11

**Authors:** Habtemu Jarso, Rameto Asu, Shumi Negawo, Kemal Ahmed, Biftu Geda, Mark Spigt

**Affiliations:** 1 Department of Public Health, School of Health Science, Madda Walabu University Shashemene Campus, Shashemene, Ethiopia; 2 Negele Arsi Health Center, Negele Arsi Town, West Arsi Zone, Ethiopia; 3 Department of Nursing, School of Health Science, Madda Walabu University Shashemene Campus, Shashemene, Ethiopia; 4 Department of Family Medicine, Faculty of Health, Medicine and Life Science, Maastricht University, Maastricht, The Netherlands; Debre Markos University, ETHIOPIA

## Abstract

**Background:**

Despite progress in reducing the time from HIV diagnosis to treatment initiation, achieving sustained viral suppression remains a major public health challenge among people living with HIV in Ethiopia. However, evidence on viral suppression and its predictors during the test-and-treat era remains limited.

**Objective:**

This study aimed to assess the rate and predictors of viral suppression among adolescents and adults living with HIV during the test-and-treat era, January 2018 to December 2022, Shashemene City, Ethiopia.

**Methods:**

A retrospective cohort study was conducted among 414 patients aged 15 years and older who initiated ART between January 2018 and December 2021. Patients were selected using a proportional stratified random sampling technique and followed for a maximum of two years in public health facilities in Shashemene City. Kaplan-Meier was used to estimate the median time to viral suppression, and Cox proportional hazards regression identified predictors of suppression.

**Results:**

Of the 414 patients, 290 (70.05%; 95% CI: 65.64–74.46) achieved viral suppression by the end of the follow-up period (December 2022). The rate of viral suppression was 6.68 per 100 person-months (95%CI: 5.95–7.49), and the median time to suppression was 9.33 months (95%CI: 8.55–10.11). Rapid ART initiation was associated with twofold higher likelihood of early suppression (aHR = 2.05, 95%CI: 1.58–2.67) compared to delayed initiation. Undernourished patients had 46% lower chance of early suppression (aHR = 0.54, 95%CI: 0.36–0.81), and those who were ambulatory/bedridden at baseline had an 87% lower chance (aHR = 0.13; 95%CI: 0.07–0.27) than working patients. Not good adherence lowered suppression by 36% (aHR = 0.64; 95%CI: 0.41–0.99).

**Conclusion and recommendation:**

The viral suppression rate was well below the UNAIDS target of 95%. Strengthening rapid ART initiation, adherence and providing nutritional support are essential to improve outcomes. Lower suppression rates among ambulatory/bedridden patients could partly reflect additional health challenges influencing treatment response.

## Introduction

Human Immunodeficiency Virus (HIV) continues to be a global public health problem, with an estimated 38.4 million people living with the virus as of 2021 [1,2]. Ethiopia, one of the 25 countries with the highest number of new HIV infections worldwide, has made significant efforts to combat the epidemic [3], with approximately 0.61 million people living with HIV as of 2022 [4].

Antiretroviral therapy (ART), introduced in 1987, has significantly reduced HIV-related morbidity, mortality, and transmission [5]. ART helps reduce viral load [5,6] and its success has transformed HIV into a chronic condition, making progression to AIDS rare [6]. Since 2016, the goal of ART has been to achieve an undetectable viral load, which also prevents sexual transmission of HIV, promoting the “undetectable equals untransmittable” (U = U) campaign [7,8]. By mid-2021, 28.7 million people were receiving ART worldwide [9], with viral load testing being the preferred method to monitor treatment success [10]. Achieving and sustaining viral load suppression, the third 95 in the updated UNAIDS 95-95-95 targets introduced in December 2020, is critical for accelerating global efforts to end AIDS by 2030 [2,6,11–13].

To improve ART effectiveness, the WHO recommended rapid ART initiation within seven days of diagnosis, which has been shown to boost retention in care and treatment outcomes [10,14–17]. UNAIDS pointed out that the introduction of same-day ART initiation was one of the most important cornerstones for the successful achievement of the 90-90-90 targets [18]. However, studies on the effects of rapid ART initiation have produced inconsistent results.

International observational [19], interventional [20–22], and systematic review [23] studies conducted among patients aged 15 years and above demonstrated that early ART initiation improves morbidity [19–21], viral suppression [22,23], and retention [23], while reducing loss to follow-up (LTFU) [19] and mortality [23]. African studies showed mixed outcomes: some interventional studies reported improved retention [24–28] and viral suppression [24–26,29–31], as well as reduced morbidity [29,32,33] and mortality [29,32]. Others, however, reported no improvement in outcomes [34,35], higher LTFU [35], or early retention problem [36]. Observational studies revealed both higher retention [37,38] and increased LTFU [39,40], lower rates of retention [41–43], higher viral suppression [44], and no improvement in outcomes [45]. Ethiopian studies, all of which were observational, reported lower retention [15] or higher LTFU [46], lower adherence [47], and lower viral suppression [48], but reduced morbidity [49] and mortality [50,51].

Ethiopia began offering free ART in 2005 [52], and by December 2022, 454,257 people were receiving it [53]. It introduced “Immediate” ART initiation in 2016 [15,17,50,54] and same-day ART initiation in 2017 [14,52,55,56], but there is limited evidence on its effectiveness in routine clinical settings. As of 2022, Ethiopia’s viral load suppression was 91% according to the report by the Ethiopian Public Health Institute (EPHI) [57]. A recent comparative study from Addis Ababa also reported an overall adolescent viral suppression of 88.3%, with 92.4% among participants in the Operation Triple Zero (OTZ) group and 84.3% in the regular ART group [58]. In contrast, a recent systematic review and meta-analysis of studies conducted on different population and regimen before and during the test-and treat era reported 71% and constantly decreasing trend from 79% in 2019 to 69% in 2023 [59]. This discrepancy highlights the need for further assessment of the rate of viral load suppression and its predictors among patients initiating ART in the test-and-treat era. Unlike earlier Ethiopian studies, the current study focused exclusively on viral load suppression during the test-and-treat era and evaluated the effect of rapid ART initiation on viral load suppression. Moreover, the study included all patients who initiated ART during the study period, regardless of their enhanced adherence counseling (EAC) status.

Andersen’s model [60–63] was used to provide a theoretical basis for understanding variations in viral suppression among adolescents and adults living with HIV in Ethiopia. Socio-demographic characteristics, residence, disclosure status, and partner’s sero-status were considered predisposing factors influencing individuals’ motivation and capacity to engage in HIV care. Enabling factors included treatment related conditions such as facility level, baseline adherence, prophylaxis use and rapid ART initiation, which facilitate or constrain access to effective treatment. Need factors were clinical stage, functional status, nutritional status, opportunistic infection, and TB screening result, which reflect the health status driving the requirement for care. Viral suppression was conceptualized as the health outcome resulting from the interplay of these factors.

## Materials and methods

### Study area and setting

The study was conducted in Shashemene City, Oromia region. Shashemene City is the capital of West Arsi zone and located approximately 250 km from Addis Ababa, capital city of Ethiopia. The city had a total population of 386,447 with females comprising 49.5%. There were two public hospitals and two public health centers providing ART service in the city. About 726 patients aged 15 years and older were on ART in the city from 2018 to 2022.

Nurses/health officers that took training on National Consolidated Guidelines for Comprehensive HIV Prevention, Care and Treatment mainly do the ART initiation and follow-up. Viral load measurements are at 6 months, 12 months and then every 12 months. Following ART initiation, patients are appointed to visit ART clinic after 2 weeks, 1, 3, or 6 months, regardless of how ART was initiated (rapid or not) [52].

### Study design and population

A facility-based retrospective cohort study was conducted among randomly selected patients aged 15 years and older who initiated antiretroviral treatment between January 2018 and December 2021, with follow-up until December 2022. The study included new ART patients whose ART initiation date was documented, who had been on ART for at least 6 months and who had documented at least one viral load measurement, regardless of their enhanced adherence counseling (EAC) status.

### Sample size and sampling technique

The maximum sample size was determined using Epi-Info version 7 assuming 45% viral suppression among patients with opportunistic infection (exposed), 59.2% viral suppression among patients without opportunistic infection (unexposed) at baseline [64], 80% power, 95% confidence level and equal ratio of exposed and unexposed patients.

Patients were selected using proportional stratified random sampling. First, the sample size was proportionally allocated across four health facilities in Shashemene City that provide ART services. Next, a sampling frame of eligible patients was developed for each facility using the ART patient electronic database. Finally, patients were selected through simple random sampling using computer-generated random numbers.

### Variables

Dependent variable: Rate of viral suppression

Independent variables:

- Socio demographic characteristics (age, marital status, gender, education, occupation, religion, residence, partner’s HIV status, and disclosure status)
- Baseline clinical characteristics: WHO clinical stage, nutritional status, TB screening result, opportunistic infection (OI), functional status
- Treatment-related factors: facility level, baseline adherence to treatment, prophylaxis use [Cotrimoxazole preventive therapy (CPT), Tuberculosis preventive therapy (TPT)], rapid ART initiation

### Data collection

Data were extracted from records and the electronic database (SMART-CARE) between June 1, 2023 and July 25, 2023. Data extraction tool was adapted from the revised 2020 Ethiopian Federal Ministry of Health HIV care/ART intake and follow up forms. The formats included socio-demographic characteristics, clinical information, and treatment related information. Baseline adherence to the treatment was measured by the number of doses missed, as self-report by patients at their first appointment, when they had been on ART for one or two months.

ART care providers and data clerk staffs collected the data. One day training was given for data collectors and tool was pre-tested and revised before starting actual data collection. Principal investigator closely supervised the data collection procedure. We checked completeness and consistency of filled formats on daily basis. Ten randomly selected completed tools were cross-checked with the medical records of the patients.

### Operational definitions

**Event:** viral suppression at any point during the follow-up period between January 2018 and December 2022.

**Viral suppression**: viral load less than or equal to 1000 copies/ml or below the detection threshold using viral assays [52]

**Censored**: lost to follow-up or transferred out or switched to second-line or died during follow-up period between January 2018 and December 2022 or alive and on ART but did not achieve viral suppression at the end of follow-up (December 2022).

**Lost to follow up (LTFU)**: a patient who did not refill his/her ARV drugs for a period of one to three months after last refill appointment date [52]

**Dropped out**: a patient who did not refill his/her ARV drugs for a period of more than three months after last refill appointment date [52]

**Baseline adherence**: adherence assessed at first appointment when a patient was one or two months on ART and classified as follows:

- **Good adherence**: adherence level **greater than** or equal to **95%,** i.e., missing <2 doses out of 30 doses or missing ≤3 doses out of 60 doses [57].
- **Fair adherence**: adherence level 85%–94%, i.e., missing 2–4 doses out of 30 doses or 4–9 doses out of 60 doses [57].
- **Poor adherence**: adherence level less than 85%, i.e., missing ≥5 doses out of 30 doses or ≥ 10 doses out of 60 doses [57]
- **Not good adherence**: fair and poor adherence combined into one category for analysis

**Rapid ART initiation:** starting ART on the same day or within 7 days of knowing HIV positive result [14,52].

**Nutritional status:** it was assessed using either body mass index (BMI) or mid-upper arm circumference (MUAC) and classified as follows:

- **Not undernourished**: BMI ≥ 18.5 kg/m^2^ for non-pregnant/non postpartum; MUAC >23 cm for pregnant/postpartum/bedridden [57]
- **Mild under nutrition**: BMI 17–18.49 kg/m^2^ for non-pregnant/non postpartum [57]
- **Moderate under nutrition**: BMI 16–16.99 kg/m^2^ for non-pregnant/non postpartum; MUAC 19–23 cm for pregnant/postpartum/bedridden [57]
- **Severe under nutrition**: BMI < 16 kg/m^2^ for non-pregnant/non postpartum; MUAC <19 cm for pregnant/postpartum; MUAC <18 cm for bedridden [57]
- **Undernourished**: mild, moderate and severe under nutrition combined into one category for analysis

### Data analysis

The data were entered into Epi-Data 3.1 and exported to SPSS version 27 and Stata version 15 for analysis. Data were cleaned and explored before data analysis. Descriptive statistics was used to summarize the data. Proportions and rates of viral load suppression and corresponding confidence intervals were calculated at different points of follow-up. The Kaplan-Meier (KM) was used to estimate the median time to viral suppression. The Cox proportional hazards regression model was used to identify the predictors of time to viral load suppression. The proportional hazards assumption was evaluated graphically using Kaplan-Meier (KM) survival curves and log-log survival plots, as well as statistically using the global test in Stata for all candidate predictors. However, survival curves of only significant predictors are shown in the Results section. In addition, the assumption was assessed using the global test in the multivariable Cox regression model. Multicollinearity among exposure variables was assessed using linear regression and evaluated using the variance inflation factor (VIF), with a VIF ≥ 10 considered indicative of potential multicollinearity (S1 Table). Effect/association modification was assessed by testing interaction terms. A statistically significant interaction was detected between baseline WHO clinical stage and baseline functional status (p-value = 0.031); therefore, this interaction term was initially included in the multivariable Cox regression model. However, the interaction was not statistically significant in the multivariable Cox regression model (p-value = 0.266), despite its biological plausibility.

We compared the global test results of the multivariable Cox regression models with and without the interaction term to inform model selection. The model without the interaction term showed no statistically significant global test results for the individual variables or for the overall model (S2 Table). In contrast, in the model including the interaction term, rapid ART initiation was statistically significant, while the overall model remained non-significant (S3 Table). Based on these results and the lack of statistical evidence for the interaction term, the model without the interaction term was retained as the final model.

Model fit was assessed using Cox-Snell residuals. The residual plot closely followed the 45-degree reference line, indicating an adequate fit of the final Cox regression model (S1 Fig). Significance of association was declared at p value < 0.05 and strength of association was reported by adjusted hazard ratio (aHR).

### Ethical considerations

Ethical clearance was obtained before data collection from the Institutional Research Ethics Review Committee (IRERC) of Madda Walabu University, Shashemene Campus (Ref. No IRERC/014/2023). The permission to use the data was also obtained from the Shashemene City Health Office and each health facility. Informed consent was waived by the ethics committee because the study involved only secondary data from patient records and did not require direct interviews. Confidentiality of information was maintained by omitting personal identifiers from data collection tool, except for medical registration number (MRN) and unique ART number (UAN). Completed tools were kept away from access by a third person except research team.

## Results

### Socio-demographic characteristics

Out of the 414 patients enrolled in the study, two-thirds [277 (66.91%)] were from Abosto Health Center and Melka Oda General Hospital. Females accounted for 272 (65.70%) of the study population. The mean age of the patients was 40.71 years (SD = 11.90), ranging from 15 to 83 years. The highest educational level attained by 317 (76.57%) patients was primary school. A total of 291 (70.29%) patients lived within the catchment area of the health facility. The partner’s HIV status was positive in 265 (64.01%) and unknown in 138 (33.33%) patients (Table 1).

**Table 1:** Socio-demographic characteristics of patients on antiretroviral treatment in public health facilities in Shashemene City, West Arsi zone, Ethiopia, Jan 2018–Dec 2022 (n = 414).

Variables	Category	Number	Percentage
**Facility name**	Abosto Health Center	150	36.23
	Melka Oda General Hospital	127	30.68
	Shashemene Comprehensive Specialized Hospital	110	26.57
	Bulchana Health Center	27	6.52
**Age of the patient**	15-24	36	8.70
	25-34	83	20.05
	35-44	151	36.47
	≥45	144	34.78
**Educational status**	No formal education	49	11.84
	Primary	317	76.57
	Secondary	32	7.73
	Diploma and above	16	3.86
**Marital status**	Married	267	64.49
	Single	139	33.57
	Widowed/Divorced	8	1.93
**Occupation**	House wife	192	46.38
	Student	100	24.15
	Farmer	88	21.30
	Daily laborer	20	4.83
	Employee	9	2.17
	Jobless	5	1.21
**Religion**	Orthodox	212	51.21
	Muslim	161	38.89
	Protestant	24	5.80
	Catholic	9	2.17
	Wakefeta	8	1.93

### Clinical and treatment related characteristics

HIV status was disclosed in 313 (75.60%) patients. ART initiation was same-day in 108 (26.09%) and 1–4 days in 81 (19.57%) patients. At baseline, 358 (86.47%) patients had a working functional status, and 348 (84.06%) were in WHO clinical stage I. Opportunistic infections were diagnosed in 66 (15.94%) patients at baseline. Cotrimoxazole and isoniazid prophylaxis were taken by 246 (59.42%) and 230 (55.56%) patients, respectively. Baseline adherence to ART was good in 349 (84.30%) patients (Table 2).

**Table 2:** Clinical and treatment characteristics of patients on antiretroviral treatment in public health facilities in Shashemene City, West Arsi zone, Ethiopia, Jan 2018 – Dec 2022 (n = 414).

Variables	Category	Number	Percentage
**Baseline Functional Status**	Working	358	86.47
	Ambulatory	51	12.32
	Bed ridden	5	1.21
**Baseline WHO Clinical Stage**	I	348	84.06
	II	46	11.11
	III	14	3.38
	IV	6	1.45
**Baseline CD4 Count**	< 200	20	4.83
	200 - 349	85	20.53
	350-500	205	49.52
	> 500	86	20.77
	Not recorded	18	4.35
**Baseline Nutritional Status**	Not undernourished	309	74.64
	Mild	45	10.87
	Moderate	41	9.90
	Severe	19	4.59
**Type of opportunistic infection at baseline**	Herpes Zoster	19	28.79
	ulcers-mouth	18	27.27
	PTB	13	19.70
	pruritic erupt	9	13.64
	Kaposi Sarcoma	4	6.06
	PCP	2	3.03
	Bacterial Pneumonia	1	1.52
**Baseline adherence to ART**	Good	349	84.30
	Fair	42	10.14
	Poor	23	5.56

### Rate of viral load suppression

A total of 414 patients were followed-up for a total of 4341.80 person-months between January 2018 and December 2022. Among these, 99 (79.84%) were censored before the end of the follow-up: 51 (51.52%) due to loss to follow-up or dropout, 28 (28.28%) were transferred out, 7 (7.07%) switched to second-line ART, and 13 (13.13%) died. The remaining 25 (20.16%) censored patients were alive and on ART but had not achieved viral suppression by the end of follow-up (December 2022. Of those censored before the end of the follow-up, 77(77.78%) were censored before their seventh month on ART (Fig 1).

**Fig 1:**
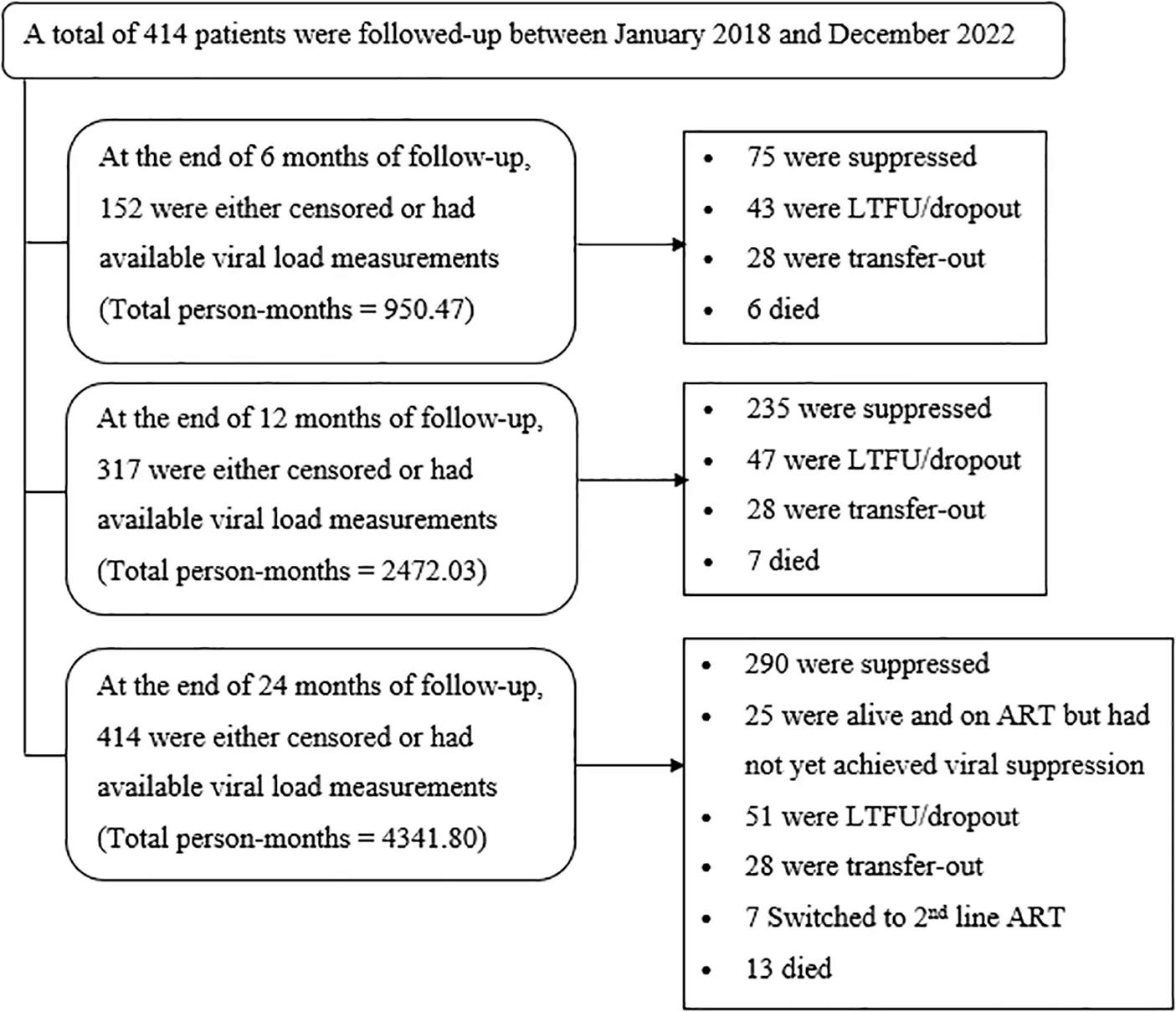
Flow diagram of follow-up and viral suppression among patients receiving ART, Shashemene City, 2018–2022.

Among the 414 patients enrolled, 75 of 152 (49.34%; 95% CI: 41.40–57.30) and 235 of 317 (74.13%, 95% CI: 69.31–78.95) patients who were either censored or had available viral load measurements achieved viral suppression at the end of 6 and 12 months of follow-up, respectively, while 290 (70.05%; 95% CI: 65.64–74.46) were virally suppressed at the end of 24 months of follow-up. The rates of viral suppression were 7.89 (95% CI: 6.29–9.89), 9.51 (95% CI: 8.37–10.80) and 6.68 (95% CI: 5.95–7.49) per 100 person-months at 6, 12 and 24 months of follow up (Fig 1). The median duration to viral suppression was 9.33 months (95% CI: 8.55–10.11) (Fig 2).

**Fig 2:**
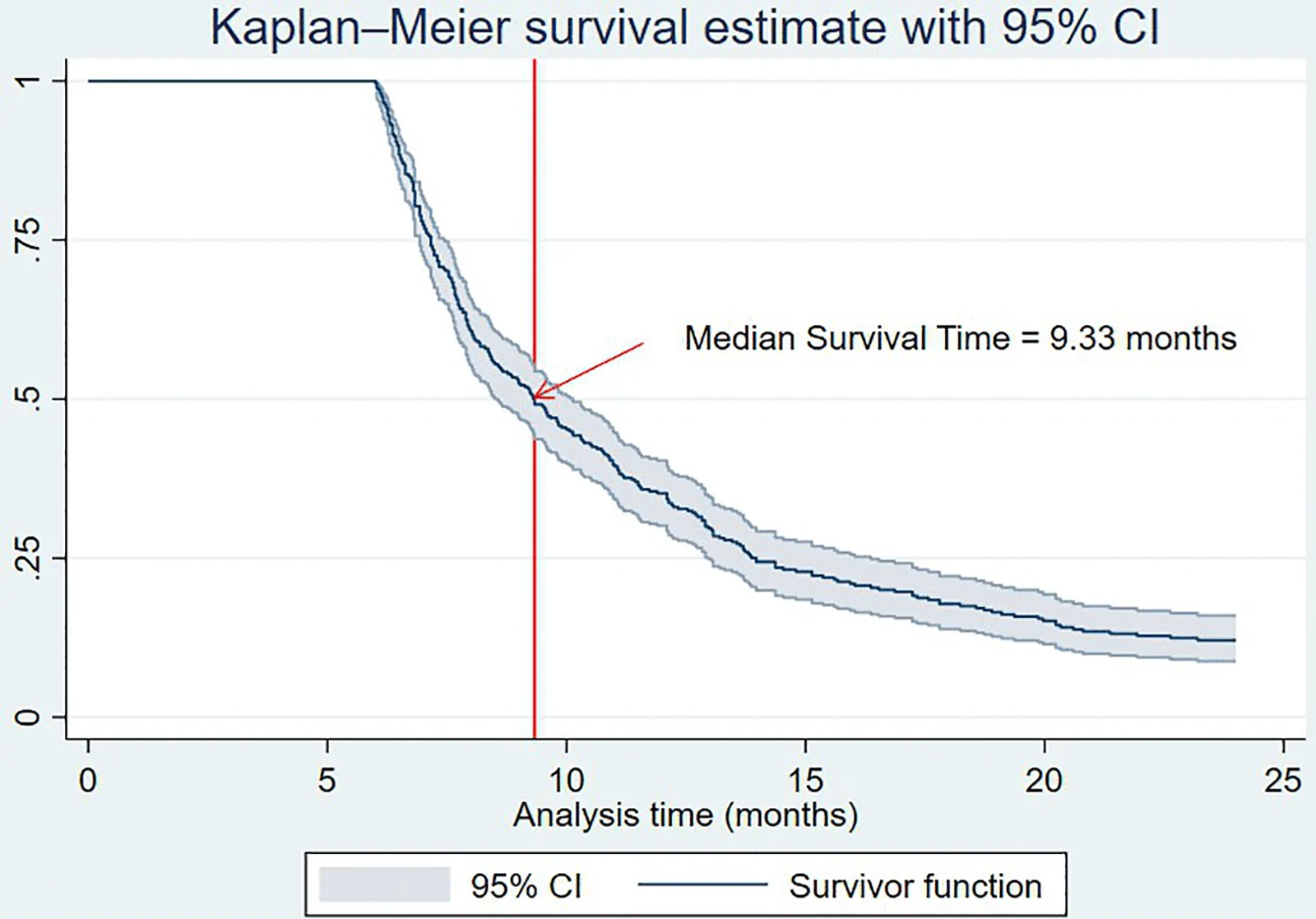
Kaplan-Meier estimate of time to viral suppression among patients receiving ART, Shashemene City, 2018–2022.

Among the 171 patients who initiated ART on the same day as diagnosis, 45 of 67 (67.16%; 95% CI: 55.92–78.41) and 130 of 152 (85.53%; 95% CI: 79.93–91.12) patients who were either censored or had available viral load measurements achieved viral suppression at 6 and 12 months of follow-up, respectively, while 144 (84.21%; 95% CI: 78.75–89.68) were virally suppressed at 24 months.

### Predictors of viral load suppression

In bivariate Cox proportional hazards regression, none of the socio-demographic characteristics such as gender, age group, marital status, religion, education, residence and living in catchment area was candidate for multivariable regression at p-value less than 0.25. Variables such as level of facility, rapid ART initiation, baseline nutritional status, baseline functional status, baseline WHO clinical stage, baseline TB screening result, baseline opportunistic infections, adherence to ARV drugs, and disclosure status were candidate and/or significant predictors of viral load suppression. However, facility level and disclosure status did not fulfill proportional hazard assumption and baseline opportunistic infection had multicollinearity (VIF = 12.2). In the multivariable Cox regression model, rapid ART initiation, baseline nutritional status, functional status, and ART adherence were the only predictors of viral suppression (Table 3).

**Table 3:** Bivariate and Multivariable Cox proportional hazards regression of clinical and treatment characteristics of patients on antiretroviral treatment in public health facilities in Shashemene City, West Arsi Zone, Ethiopia, Jan 2018–Dec 2022 (n = 414).

Factors	Category	Events	Censored	cHR (95% CI)	aHR (95% CI)	p-value
**Rapid ART initiation**	Yes	177	30	2.73 (2.12,3.50)	2.05 (1.58,2.67)	< 0.001
	No	113	94	1	1	
**Baseline nutritional status**	Undernourished	45	60	0.18 (0.13,0.26)	0.54 (0.36,0.81)	0.003
	Not undernourished	245	64	1	1	
**Baseline functional status**	Ambulatory/ Bedridden	15	41	0.07 (0.04,0.13)	0.13 (0.07,0.27)	<0.001
	Working	275	83	1	1	
**Baseline WHO clinical stage**	Stage III/IV	7	13	0.35 (0.17,0.75)	2.34 (0.92,5.97)	0.075
	Stage I/II	283	111	1	1	
**Baseline TB screening result**	Positive	10	24	0.19 (0.10,0.37)	0.91 (0.40,2.06)	0.826
	Negative	280	100	1	1	
**Baseline ART adherence**	Not good	29	36	0.35 (0.24,0.52)	0.64 (0.41,0.99)	0.049
	Good	261	88	1	1	

In multivariable Cox proportional hazards regression, rapid ART initiation, baseline nutritional status, and baseline functional status were the only factors remained significant predictors of viral load suppression. Baseline adherence was marginally significant. Patients who initiated ART rapidly (within one week of HIV diagnosis) achieved viral load suppression at a significantly faster rate than those who initiated ART later. Specifically, rapid ART initiation was associated with a twofold higher chance of earlier viral load suppression (AHR = 2.05; 95% CI = 1.58–2.67; p < 0.001). The Kaplan-Meier survival curves demonstrated a clear separation between the two groups with the steeper decline in the survival curve for patients who rapidly initiated ART. (Fig 3).

**Fig 3:**
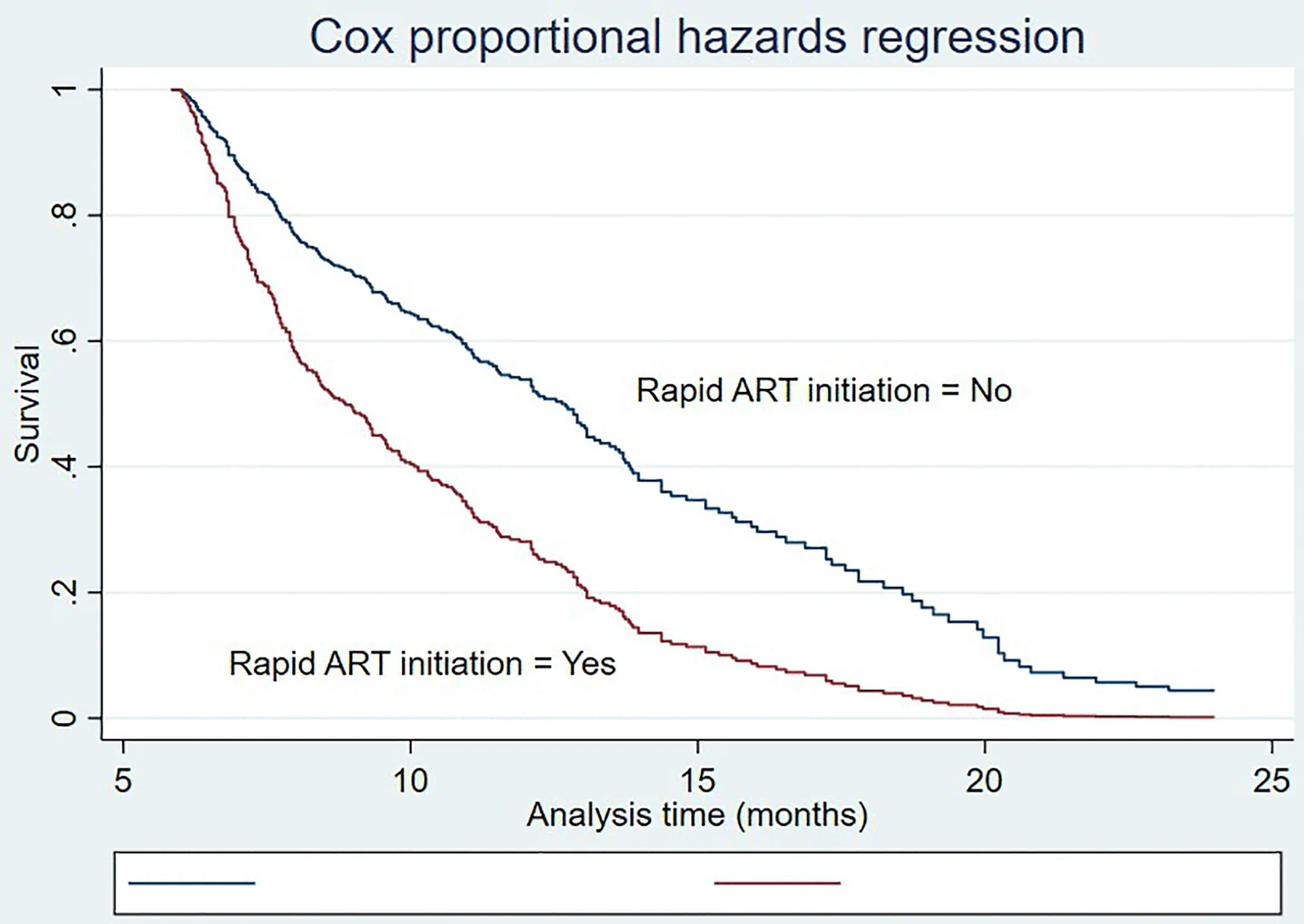
Kaplan-Meier curves of time to viral suppression by ART initiation timing, Shashemene City, 2018–2022.

Patients who were undernourished at baseline had a significantly lower rate of achieving viral load suppression compared with patients who were not undernourished. Specifically, patients who were undernourished at baseline had a 46% lower chance of earlier viral load suppression (AHR = 0.54; 95% CI: 0.36–0.81, p = 0.003). The Kaplan-Meier survival curves demonstrated a clear separation between the two groups, with undernourished patients experiencing delayed viral load suppression throughout the follow-up period (Fig 4).

**Fig 4:**
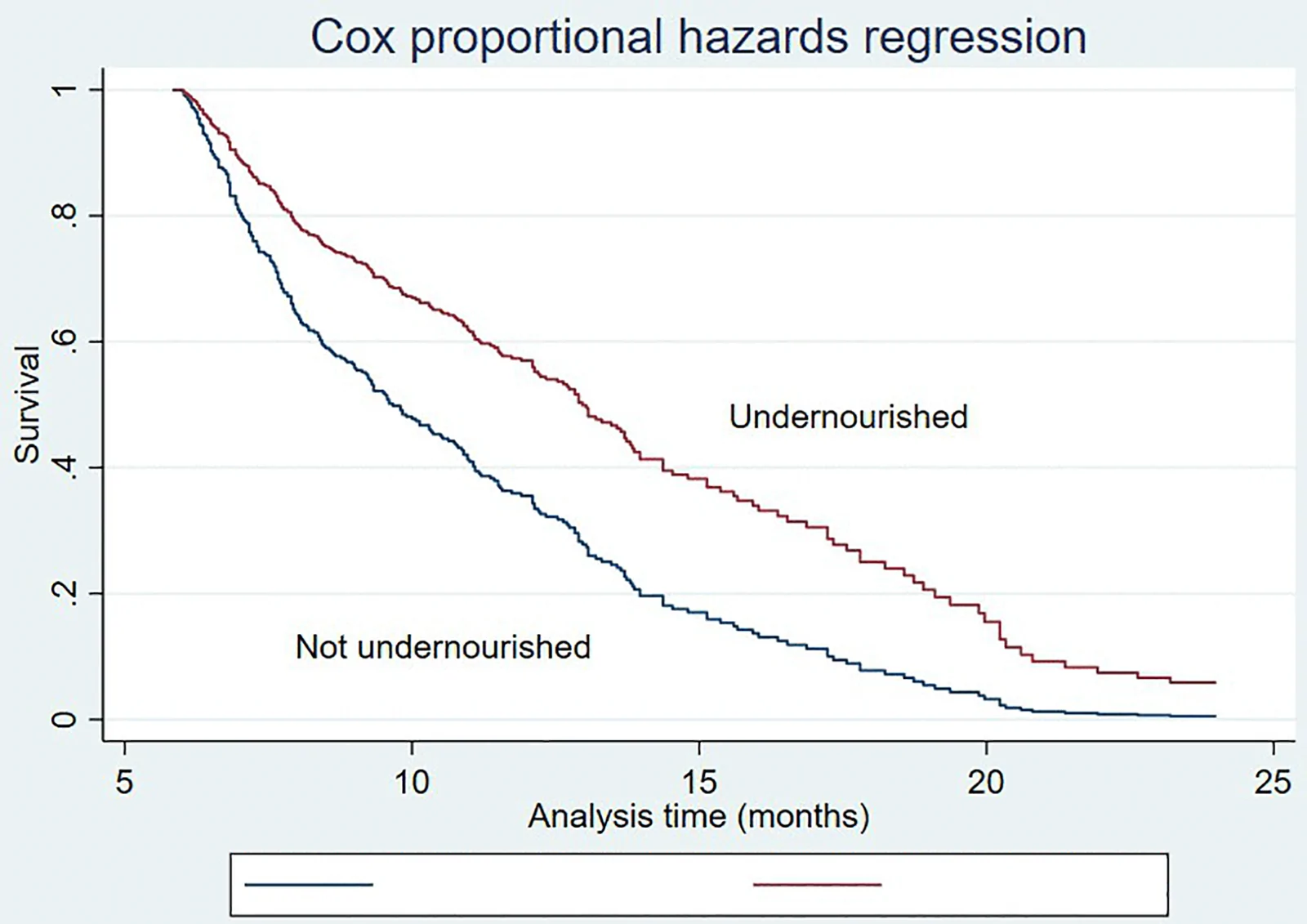
Kaplan-Meier curves of time to viral suppression by baseline nutritional status, Shashemene City, 2018–2022.

Patients who had an ambulatory or bedridden functional status at baseline had an 87% lower chance of earlier achieving viral load suppression than those with a working functional status (AHR = 0.13; 95% CI: 0.07–0.27; p < 0.001). The Kaplan-Meier survival curves demonstrated a marked difference in time to viral load suppression between the two groups, with ambulatory/bedridden patients experiencing substantially delayed viral load suppression throughout the follow-up period (Fig 5).

**Fig 5:**
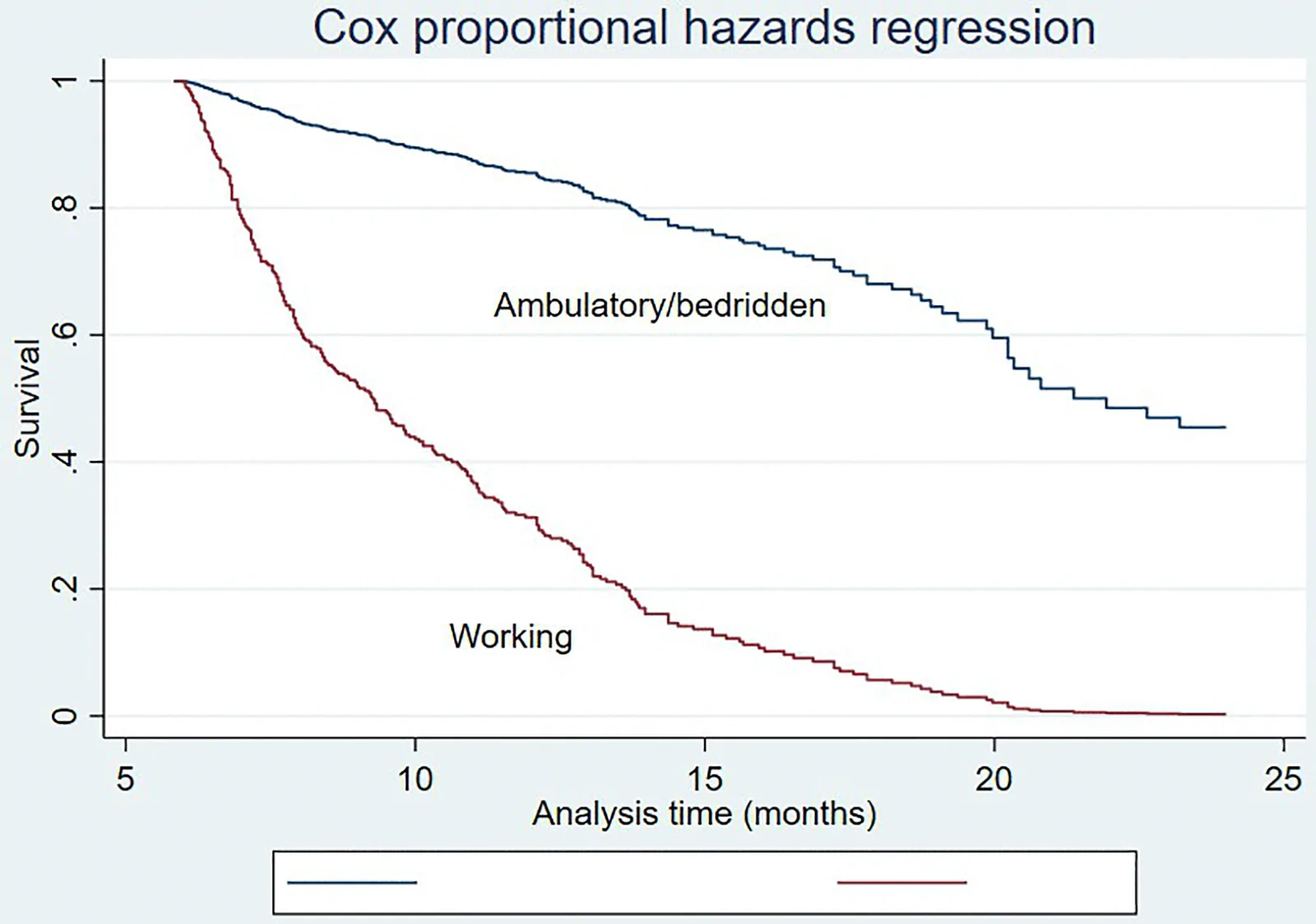
Kaplan-Meier curves of time to viral suppression by baseline functional status, Shashemene City, 2018–2022.

Patients with not good baseline ART adherence had a 36% lower chance of achieving earlier viral load suppression compared with patients who had good ART adherence at baseline (AHR = 0.64; 95% CI: 0.41–0.99; p = 0.049). The Kaplan-Meier survival curves demonstrated that patients with not good adherence experienced delayed viral load suppression throughout the follow-up period compared with those who maintained good adherence (Fig 6).

**Fig 6:**
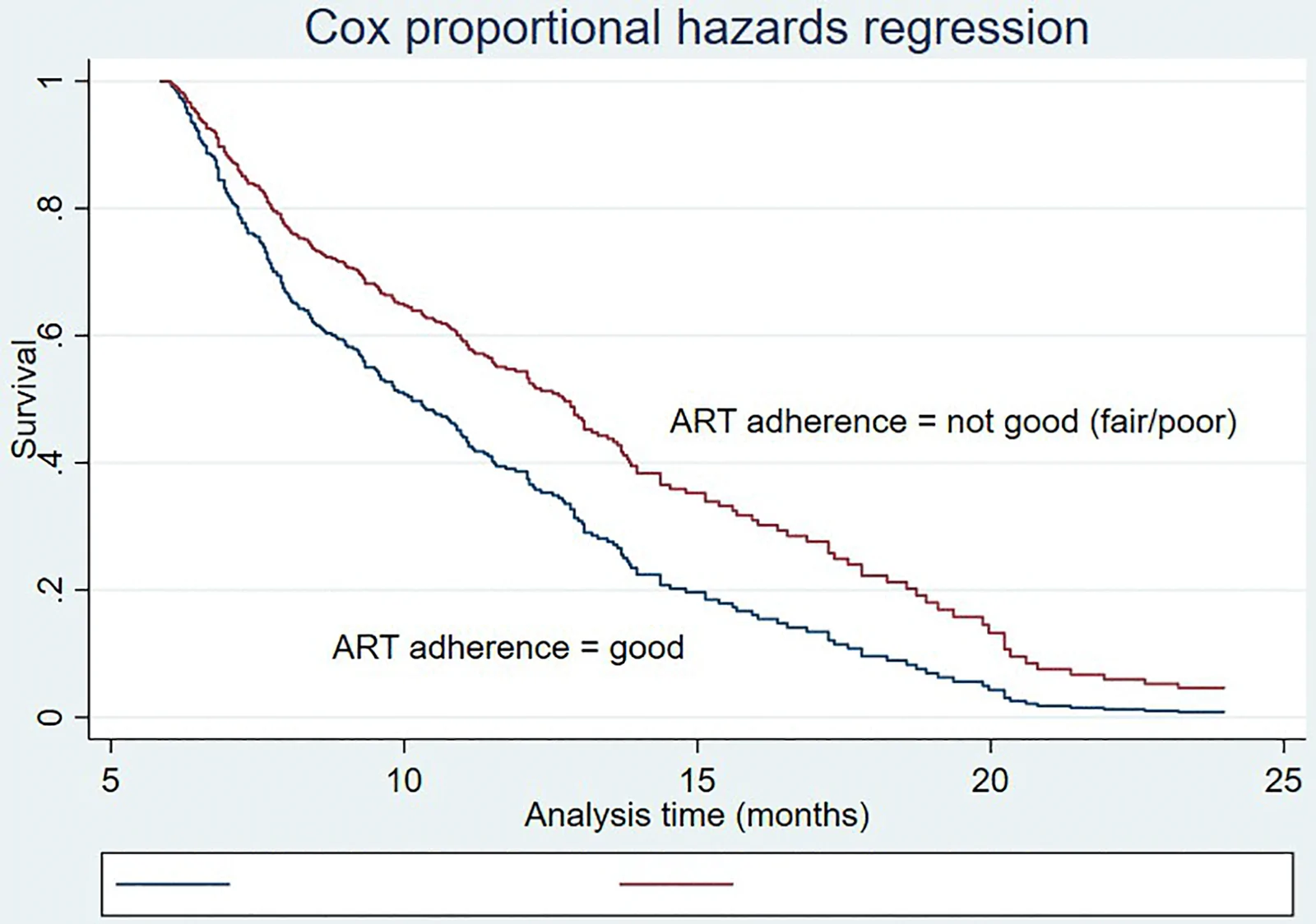
Kaplan-Meier curves of time to viral suppression by baseline ART adherence, Shashemene City, 2018–2022.

## Discussion

Achieving viral suppression is essential in HIV treatment for both improving individual health outcomes and reducing transmission at the community level. We conducted a retrospective cohort study to estimate the rate of viral suppression and identify its predictors. Our findings revealed that 70.05% of patients achieved viral suppression by the end of the follow-up period, with an incidence rate of 6.68 per 100 person-months. The median time to viral suppression was 9.33 months. These results showed that there is a progress towards the global target of 95% by 2030. However, the proportion in the current study falls far short of the 2030 global target indicating urgent need to double efforts to achieve it. Rapid ART initiation, baseline nutritional status, baseline functional status and adherence were independent predictors of viral suppression.

The proportion of viral suppression in our study was consistent with findings from previous Ethiopian studies conducted during the test-and-treat era [65]. It was also comparable to the pooled estimate reported in a recent systematic review of studies conducted in Ethiopia among different populations and using different ART regimens [59]. Similarly, the pooled estimate from five studies among adults receiving first-line ART during the test-and-treat era was consistent with our findings [66–70]. Among same-day ART initiators, the proportion of viral suppression in our study was also comparable to findings from previous Ethiopian studies, including a cross-sectional study assessing suppression at 12 months [56] and a retrospective cohort study [48].

Our findings therefore complement previous Ethiopian evidence by providing longitudinal data on viral suppression over the first two years of treatment among patients who initiated ART exclusively during the test-and-treat era. Unlike studies that specifically focused on same-day ART initiators, our study included patients regardless of the interval between HIV diagnosis and ART initiation, thereby providing a broader assessment of viral suppression among patients receiving ART in routine care.

However, our findings also reveal concerning disparities compared to several other previous studies. The proportion of viral suppression in our study was significantly lower than that reported by earlier Ethiopian cross-sectional [69], retrospective [48] and prospective [68] cohort studies conducted among adults on first-line regimen in the test-and-treat era. Both the proportion and absolute rate were also considerably lower than those reported by more recent Ethiopian retrospective follow-up studies [67,71]. These differences may partly be attributed to variations in study settings, designs, durations, and the sociodemographic and clinical characteristics of the study participants. The viral suppression proportion in our study was also lower than that reported in a previous cross-sectional study [72], in which more than half of patients with high baseline viral loads (>1000 copies/mL) defaulted and were not re-tested. This may suggest that lost to follow-up of patients with high viral loads could have led to an overestimation of viral suppression in that study. Furthermore, our proportion was lower than that reported in a comparative cross-sectional study among adolescents enrolled in the Operation Triple Zero (OTZ) and regular ART programs in Addis Ababa [58], possibly due to differences in socio-demographic characteristics and the beneficial effects of the OTZ intervention among adolescents.

Among patients who initiated ART on the same day as their diagnosis, the viral suppression proportion at six and twenty-four months in our study was lower than the findings of previous Ethiopian cross-sectional study [56]. The median time to viral suppression in our study was also longer than that reported in one Ethiopian study [71], but consistent with findings from another study [67]. These discrepancies may partly be explained by the clinical characteristics of study populations and differences in the proportion of loss to follow-up (LTFU), transfer out (TO) and deaths, as retention in care plays critical role in the achievement of early viral suppression [73].

One key factor that made a difference in patients’ viral suppression was the timing of ART initiation. Patients who rapidly initiated ART were twice as likely to achieve earlier viral load suppression as patients who did not. Our results were consistent with previous studies that reported significantly higher viral load suppression in patients who started ART rapidly compared to those who started ART one week after diagnosis [19,56,70]. This finding adds to the growing body of evidence supporting the implementation of same-day ART initiation and underscores the importance of minimizing delays in treatment initiation. However, some studies have reported contrasting results, suggesting that early ART alone may not always guarantee better outcomes [31,41,48].

Nutritional status also played a role in shaping treatment outcomes. Undernourished patients were almost half as likely to achieve earlier viral load suppression as patients who were not undernourished. This was consistent with the findings of previous studies which reported that patients who were undernourished had lower [69,74], and well-nourished patients had higher [59,65], chances of achieving earlier viral load suppression. This result underscores the importance of adequate nutrition in supporting immune function and optimizing treatment response, and it reinforces the need to provide nutritional support even for patients with mild malnutrition, as poor nutrition can weaken the immune system and promote viral replication [75].

Another factor influencing early viral load suppression was the patients’ baseline functional status. Patients with limited mobility (ambulatory or bedridden) had approximately one-eighth the chance of achieving early viral load suppression compared to those with a baseline working functional status. This finding suggests that individuals with physical impairment at baseline could have additional health issues or factors that influence drug response.

In contrast, WHO clinical stage was only marginally associated with early viral load suppression. This may be because a patient can be ambulatory or bedridden even at early WHO clinical stage, for example, due to coexisting illness, malnutrition or opportunistic infections. Conversely, some stage III/IV patients may still be working (physically active) if they have mild symptoms. Functional status is likely more proximal to treatment success, as bedridden or ambulatory patients may struggle with attending clinic visits, swallowing mediations, or recovering immunity. Contrary to our results, earlier studies reported that patients with early-stage HIV (WHO clinical stage I or II) had higher chances of achieving earlier viral load suppression [67], whereas patients with advanced stages (stage III or IV) had lower chances of earlier viral load suppression [65,71]. Our results did not reach statistical significance, possibly because patients with advanced stages accounted for only five percent of the study population. Future research should investigate the role of comorbidities to better understand the mechanisms underlying this discrepancy.

Baseline adherence to ART, measured by self-report, was also weakly associated with viral load suppression, where patients whose adherence was not good had lower chances of viral load suppression. The lack of strong statistical significance may reflect limitations of self-reported adherence, which can overestimate actual medication-taking behavior. Similar findings have been reported in previous studies, where patients with poor adherence had significantly lower chance of viral suppression [69,71,74], and those with good adherence had higher chances [65,68].

Despite these insights, our study has some limitations. Because baseline viral load was not routinely measured in most patients care, we were unable to account for its potential influence on the time to viral suppression. Prior studies [59,64,65,68] have shown that baseline viral load was significantly associated with time to viral load suppression. Additionally, adherence was assessed through self-reported missed doses, which may have led to an overestimation of good adherence due to social desirability bias.

## Conclusions

Despite the progress made, our findings highlight the need for more targeted interventions. Same-day ART initiation should be prioritized through strengthened counseling and system support. Patients who delay ART initiation require closer follow-up to prevent treatment delays. Nutritional support should be expanded not only for those with severe malnutrition but any mildly undernourished patients. Furthermore, adherence support should be enhanced for all patients, particularly those with limited functional status, regardless of WHO clinical stage, as they may otherwise experience delayed viral suppression.

Ultimately, the path to viral suppression is not the same for every patient. But by understanding the factors that influence this journey, we can take meaningful steps to close the gap and move closer to the global 95% target, improving both individual and public health outcomes. We recommend future prospective studies that control for baseline viral load, provide more precise estimates of the time to viral load suppression, and include objective measures of treatment adherence.

## Supporting information

S1 Fig Cox-Snell residual plot assessing the goodness of fit of the Cox proportional hazards model. (TIF)

S1 Table Results of multicollinearity assessment using the variance inflation factor (VIF). (DOCX)

S2 Table Global test results of multivariable Cox regression for the model without the interaction term. (DOCX)

S3 Table Global test results of multivariable Cox regression for the model with the interaction term. (DOCX)

S1 File This file contains the anonymized dataset extracted from patient medical records and used to generate the results reported in the manuscript. (CSV)

## Abbreviations

AIDS: acquired immunodeficiency syndrome
aHR: adjusted hazard ratio
ART: antiretroviral therapy
cHR: crude hazard ratio
CI: confidence interval
HIV: human immunodeficiency virus
LTFU: loss to follow-up
OI: opportunistic infection
PCP: pneumocystis carinii pneumonia
PTB: pulmonary tuberculosis
TO: transfer out
UTT: universal test and treat
VIF: variance inflation factor
WHO: World Health Organization
