# Supplementary material for: Viral suppression among adolescents and adults living with human immunodeficiency virus in Ethiopia during the test-and-treat era
Source: PLoS One. 2026 Nov 11;21(11):e0362375. doi: 10.1371/journal.pone.0362375 (PMC13754153; doi:10.1371/journal.pone.0362375)
Supplement: Supplementary file 1 [file pone.0362375.s006.pdf]

2025.05.26

Dear Editor

Thank you for having confidence in me in reviewing this manuscript titled **"Viral suppression among adults living with HIC in Ethiopia during the test -and-test era"**

This is an interesting topic which warranted to be researched.

COMMENTS:

Title: Suggest writing HIC in full.

Abstract:

Well-presented, which gave readers background of the problem, the aim, sampling technique , type of analysis, the results and the contribution were well indicated. Suggest minimising the use of "We" to every sentence. With regard to the sample the author to specify what is meant by adult patients, suggest giving age range.

Background:

The background of Viral load initiative well -given from WHO perspective.

> "To improve ART effectiveness, the WHO recommended rapid ART initiation within seven days of diagnosis, which has been shown to boost retention in care and treatment outcomes (8, 11-14).UNAIDS pointed out that the introduction of same-day ART initiation was one of the most important cornerstones for the successful achievement of the 90-90-90 targets (15). *However, studies on the effects of rapid ART initiation have produced inconsistent results. Some studies report benefits such as increased retention in care (16-24), reduced morbidity and mortality (20,25-32), and rapid and/or sustained viral suppression (17, 21-24, 29, 33-36), while others show lower adherence (37, 38), higher loss to follow-up or no improvement in outcomes (13, 24, 39-54)."*

Suggest if the authors can indicate those studies in International and African context, by indicating the type of studies, the participants and the findings. Presentation to be funnel type ending with where the study was conducted, or the problem was identified. Which theory underpinned this study, this need to be indicated.

Open Rubric

Methods and Materials:

Well- presented, with elaboration of the setting, sampling with final selection of simple random sampling and where the information was found being from the registers of facilities. **The sample was adult patient. and the concern is of 15 years, can 15 years old being a teenager be regarded as an adult? This is a serious concern. The authors to give an answer to this concern, looking also at ethical implications**. Independent and dependent variables guides were indicated. Key concepts were well-defined. Data collectors were used and were trained, and the tool was pre-tested for validity. Data was analysed descriptively. The Kaplan-Meier was used. **Suggest that logic regression to be considered based on the indicated variables.**

Ethical considerations:

Elaboratively tabulated.

Results:

Table 1 Sociodemographic details. The age-range of from 15 years is a concern. Refer to comments in methods and materials. Otherwise, the results were well-presented although in the analysis of logic regression was not mentioned in data analysis section. I have commented about that in data analysis section. Ensure consistency.

Discussion

The results were discussed however**, they need to be supported with other studies. Limited information was given in this regard have noted author Chachu as cited.**

Conclusion

Well- presented, with the final results and its contribution. Suggest that authors can indicate future studies.

Technical. The authors to adhere to journal author Guidelines.

RECOMMENDATION:

This is an interesting study which is worth to be published. Acceptance is recommended after attendance of the indicate minor corrections.

REVIEWER:

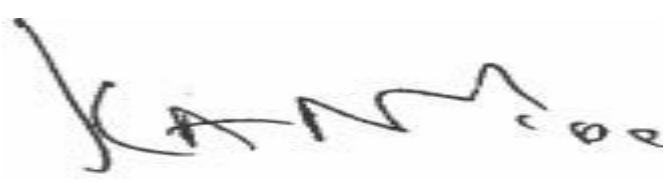

Prof KA MABOE

Department of Health Studies

University of South Africa

Emails maboeka@unisa.ac.za and maboekefiloe@gmail.com
