# Supplementary material for: Viral suppression among adolescents and adults living with human immunodeficiency virus in Ethiopia during the test-and-treat era
Source: PLoS One. 2026 Nov 11;21(11):e0362375. doi: 10.1371/journal.pone.0362375 (PMC13754153; doi:10.1371/journal.pone.0362375)
Supplement: Supplementary file 2 [file pone.0362375.s002.docx]

**S1 Table**. Results of multicollinearity assessment using the variance inflation factor (VIF)
