# Supplementary material for: Viral suppression among adolescents and adults living with human immunodeficiency virus in Ethiopia during the test-and-treat era
Source: PLoS One. 2026 Nov 11;21(11):e0362375. doi: 10.1371/journal.pone.0362375 (PMC13754153; doi:10.1371/journal.pone.0362375)
Supplement: Supplementary file 3 [file pone.0362375.s003.docx]

**S2 Table**. Global test results of multivariable Cox regression for the model without the interaction term
