# Supplementary material for: Viral suppression among adolescents and adults living with human immunodeficiency virus in Ethiopia during the test-and-treat era
Source: PLoS One. 2026 Nov 11;21(11):e0362375. doi: 10.1371/journal.pone.0362375 (PMC13754153; doi:10.1371/journal.pone.0362375)
Supplement: Supplementary file 5 [file pone.0362375.s007.docx]

**Reviewer’s Report**

**Manuscript Title:** Viral suppression among adults living with HIV in Ethiopia during the test-and-treat era

**Article Type:** Research Article

**General Assessment**

This manuscript addresses a timely and important issue in HIV care in Ethiopia—the rate and predictors of viral suppression in the test-and-treat era. The study design is appropriate, analyses are rigorous, and the findings have clear programmatic relevance. However, the manuscript would benefit from greater clarity in its presentation, a more concise narrative in some sections, and stronger contextualization of findings within Ethiopia’s HIV program landscape. Below are my detailed comments.

**Major Comments**

**1. Abstract**

- The phrase “further improvements are needed to achieve the End HIV epidemic target of 95% suppressed viral load” is vague consider rephrasing to specify Ethiopia’s performance gap.
- The abstract is well-structured but somewhat speculative in its conclusion. The claim that higher suppression rates among advanced-disease patients “may reflect more intensive management” should either be toned down or moved to the discussion.
- Results should consistently present both percentages and absolute numbers (e.g., “290 [70.1%] of 414 clients”).

**2. Introduction**

- Change the **Background** to **Introduction** as this section is the summary of the whole introduction
- The introduction provides a broad overview of HIV epidemiology and treatment goals but could be more concise. There is repetition regarding UNAIDS 90-90-90 and 95-95-95 targets.
- The knowledge gap needs to be sharpened earlier. Ethiopia’s current national viral suppression rates (from DHS, EPHI, or UNAIDS reports) should be explicitly cited to establish context.
- Please clarify how this study adds value compared to recent Ethiopian studies (e.g., Belete et al. 2024; Chirnet et al. 2024; Erjino et al. 2023).

**3. Methods**

- The sampling procedure needs clarification. It appears stratified sampling was first used at the facility level followed by simple random sampling within facilities. Please clarify.
- Inclusion/exclusion criteria should be stated more explicitly. For example, patients without baseline viral load were excluded, but this is only later noted as a limitation.
- The measure of adherence (self-report at first appointment) is weak and prone to bias. This limitation should be acknowledged in the methods as well as in the discussion.
- Ethical clearance is mentioned, but the approval number is not provided—this should be added.

**4. Results**

- **In Survival analysis–based studies** like this one, it is very important to present and interpret both **events** (patients who achieved viral suppression) and **censored cases** (patients who did not achieve suppression during follow-up, were lost to follow-up, transferred out, or died).
- The results section is comprehensive, but the narrative repeats information already presented in tables. For clarity, the text should highlight only the most important findings.
- Attrition (loss to follow-up, deaths, transfer-outs) deserves more emphasis, given its potential impact on suppression rates.
- Interaction terms are briefly mentioned but not well explained. For instance, the reported combined effect of WHO stage and functional status is not clearly presented in the text. Nutritional status*Adherence - specified as Mild*Not good is not clear. Nutritional status is not classified as mild or moderate. You have to specify as undernourished or not.
- Rates of suppression (incidence rates vs. cumulative proportions) are sometimes difficult to follow. A clearer distinction between these metrics is recommended.
- Best practice
- You need to Present **KM curves** only for variables of **primary interest** (the study’s main predictors, significant or nearly significant ones).
- For non-significant predictors, a good compromise is:
  - Mention in the methods/results that KM curves were examined for all candidate predictors, but only significant (or key clinical) ones are shown.

**But in your report especially in the final model, most of the variables were not even candidate for the Multivariable Cox-regression.**

**5. Discussion**

- The discussion contextualizes findings against regional and global studies but is sometimes speculative. The statement about advanced-disease patients achieving faster suppression due to “more intensive care” should be tempered unless supported by references.
- Comparative analysis with Ethiopia-specific studies could be expanded, highlighting differences in study setting, design, or population.
- The policy implications should be stronger and more actionable for example:
  - Prioritizing same-day ART initiation through strengthened counseling and system support.
  - Expanding nutritional support not only for moderate/severe malnutrition but also for mild malnutrition.
  - Enhancing adherence support for patients at earlier stages of disease who may otherwise delay viral suppression.
- The limitations section is appropriate but could mention missing baseline viral load data more prominently, as this is a critical factor influencing suppression dynamics.

**Minor Comments**

1. Ensure consistency in reporting percentages (e.g., sometimes one decimal place, sometimes two).
2. In tables, consider reordering variables for readability (e.g., functional status and WHO stage together).
3. Improve grammar/phrasing in some areas, e.g., “clients whose viral load was determined” → “clients with available viral load measurements.”
4. Figures and tables should be self-explanatory; table captions could be expanded slightly to clarify denominators.
5. Consider condensing overly detailed background statistics (e.g., global HIV numbers) to focus more on Ethiopia.
