# Supplementary material for: Viral suppression among adolescents and adults living with human immunodeficiency virus in Ethiopia during the test-and-treat era
Source: PLoS One. 2026 Nov 11;21(11):e0362375. doi: 10.1371/journal.pone.0362375 (PMC13754153; doi:10.1371/journal.pone.0362375)
Supplement: Supplementary file 6 [file pone.0362375.s009.docx]

**Response to Reviewers**

**Manuscript Title**: Viral suppression among adults living with HIV in Ethiopia during the test -and-test era

Manuscript ID: PONE-D-25-07030

**Reviewer-1** (Prof KA MABOE; Emails: [maboeka@unisa.ac.za](mailto:maboeka@unisa.ac.za) and [maboekefiloe@gmail.com](mailto:maboekefiloe@gmail.com))

Dear Reviewer,

We would like to express our sincere appreciation to you for your valuable time, insightful comments, and constructive suggestions, which have greatly improved the quality and clarity of this manuscript. Your thoughtful feedback has helped refine all sections of our work and strengthen the interpretation of our findings. We are deeply grateful for your professional contribution to enhancing the overall scientific rigor of this work. We have responded to each comment and concern in detail below.

**Section: Title**

1. **Comment**: “Suggest writing HIV in full.”

**Response**

The authors used the abbreviation in the title to reduce word count and because HIV is very common abbreviation. Now, we have accepted the constructive comment and revised the title as follows:

**Viral suppression among adolescents and adults living with human immunodeficiency virus in Ethiopia during the test-and-treat era**

**Section: Abstract**

2. **Comment**: “Suggest minimizing the use of “We” to every sentence. With regard to the sample, the author to specify what is meant by adult patients, suggest giving age range.”

**Response**

The use of “we” has been minimized as suggested by the reviewer and the revised section of the abstract is provided below. Regarding the age range, individuals aged 15 years and above are considered adults for HIV treatment in Ethiopia. Most previous studies (especially Ethiopian studies) have also followed this classification, although some defined adults as individuals aged 18 years and above. According to WHO definition, the age group 10-19 years is defined as adolescents. Therefore, in our study, we revised the title and study population to “adolescent and adult patients.” However, in other sections of the manuscript, we preferred the term “patients” to avoid unnecessarily repeating “adolescent and adults” throughout, which could be monotonous for the readers.

**Abstract**

**Methods:** A retrospective cohort study was conducted among 414 patients aged 15 years and older who received antiretroviral therapy (ART) between January 2018 and December 2022. Patients were selected through a proportional stratified random sampling technique, and followed for two years in public health facilities in Shashemene City. The median survival time to viral load suppression was estimated using the Kaplan-Meier survival analysis. Predictors of time to viral suppression were identified using Cox proportional hazards regression.

**Study Design and Population**

A facility-based retrospective cohort study was conducted among randomly selected patients aged 15 years and older who initiated antiretroviral treatment between January 2018 and December 2021, with follow-up until December 2022. The study included new ART patients whose ART initiation date was documented, who had been on ART for at least 6 months and who had documented at least one viral load measurement, regardless of their enhance adherence counseling (EAC) status.

**Section: Background**

3. **Comment**: “Suggest if the authors can indicate those studies in International and African context, by indicating the type of studies, the participants and the findings. Presentation to be funnel type ending with where the study was conducted, or the problem was identified. Which theory underpinned this study, this need to be indicated”.

**Response**:

Dear Reviewer,

We appreciate this insightful comment, which helped us improve the way we summarize findings from previous studies. In response, we carefully reviewed each study to extract key information including location and period, study design, population characteristics and main findings (**see attached table**). We then synthesized these details and summarized the reviewed studies as follows:

International **observational (1), interventional (2-4),** and systematic review (5) studies conducted among adults aged 15 years and above demonstrated that early ART initiation improves morbidity (1-3), viral suppression (4, 5), and retention (5), while reducing loss to follow-up (LTFU) (1) and mortality (5). African studies showed **mixed outcomes:** some interventional studies reported improved retention (6-10) and viral suppression (6-8, 11-13), as well as reduced morbidity (11, 14, 15) and mortality (11, 14). Others, however, reported no improvement in outcomes (16, 17), higher LTFU (17), or early retention problem (18). Observational studies revealed both higher retention (19, 20) and increased **LTFU (21, 22), lower rates of retention (23-25),** higher viral suppression (26), **and** no improvement in outcomes (27). Ethiopian studies, all of which were observational, reported lower retention (28, 29) or higher LTFU (30), lower adherence (31), and lower viral suppression (32), but reduced morbidity (33) and mortality (34, 35).

Regarding the theoretical framework underpinning this study, we have added the following text to the end of the *Introduction* section of the manuscript.

Andersen’s model (57-60) was used to provide a theoretical basis for understanding variations in viral suppression among adolescents and adults living with HIV in Ethiopia. Socio-demographic characteristics, residence, disclosure status, and partner’s sero-status were considered predisposing factors influencing individuals’ motivation and capacity to engage in HIV care. Enabling factors included treatment related conditions such as facility level, baseline adherence, prophylaxis use and rapid ART initiation, which facilitate or constrain access to effective treatment. Need factors were clinical stage, functional status, nutritional status, opportunistic infection, and TB screening result, which reflect the health status driving the requirement for care. Viral suppression was conceptualized as the health outcome resulting from the interplay of these factors.

**Section: Methods and Materials**

4. **Comment**: “The sample was adult patient. The concern is of 15 years, can 15 years old being a teenager be regarded as an adult? This is a serious concern. The authors to give an answer to this concern, looking also at ethical implications.” “The Kaplan-Meier was used. Suggest that logic regression to be considered based on the indicated variables.”

**Response**

We have redefined our study population as “adolescents and adults,” and have revised the manuscript accordingly, as described above. Regarding the ethical considerations, our study involved secondary data analysis and no direct interviews were conducted with patients. Confidentiality was ensured through the use of anonymous data extraction, as detailed in the *Ethical Consideration* section of the manuscript.

Regarding our choice of Kaplan-Meier and Cox regression analyses over logistic regression, our primary interest was not only in whether viral suppression occurred, but also in the time taken to achieve viral suppression. In addition, eligible patients (those who had been on ART for at least 6 months) were followed for unequal lengths of time, and accounting for these time differences is essential for accurate analysis. The Kaplan–Meier method and Cox proportional hazards model appropriately address these time-to-event characteristics. Furthermore, previous studies conducted in Ethiopia have employed similar analytical approaches, facilitating meaningful comparison of findings across studies.

**Section: Results**

5. **Comment**: Table 1 Sociodemographic details. The age-range of from 15 years is a concern. Refer to comments in methods and materials. Otherwise, the results were well-presented although in the analysis of logic regression was not mentioned in data analysis section. I have commented about that in data analysis section. Ensure consistency.

**Response**

We have redefined our study population as “adolescents and adults,” and revised the manuscript accordingly. In addition, we have explained our rationale for choosing Kaplan-Meier and Cox regression analyses instead of logistic regression, as outline above.

**Section: Discussion**

6. **Comment:** “The results were discussed. However, they need to be supported with other studies. Limited information was given in this regard have noted author Chachu as cited.”

**Response:** We have thoroughly revised the Discussion section by expanding the comparative analysis with Ethiopia-specific studies and highlighting the differences in study settings, design, or population characteristics.

**Section: Conclusion**

7. **Comment:** “Suggest that authors can indicate future studies.”

**Response:**

We have added the following text to the last paragraph of *Conclusion* section of the manuscript:

We recommend future prospective studies that control for baseline viral load, provide more precise estimates of the time to viral suppression, and include objective measures of treatment adherence.

**Section: Technical**. The authors to adhere to journal author Guidelines.

8. **Comment**: “The authors to adhere to journal author Guidelines.”

**Response**: We have revised the manuscript in accordance with the PLOS ONE manuscript preparation guidelines to the best of our knowledge.

**Reviewer-2 (**anonymous)

Dear Reviewer,

We would like to express our sincere appreciation to you for your valuable time, insightful comments, and constructive suggestions, which have greatly improved the quality and clarity of this manuscript. Your thoughtful feedback has helped refine all sections of our work and strengthen the interpretation of our findings. We are deeply grateful for your professional contribution to enhancing the overall scientific rigor of this work. We have responded to each comment and concern in detail below.

**Major comments**

**Section: Abstract**

- **Comment**: “The phrase “further improvements are needed to achieve the End HIV epidemic target of 95% suppressed viral load” is vague consider rephrasing to specify Ethiopia’s performance gap”

**Response:** We have revised the Background section of abstract as follows:

Despite progress in reducing the time from Human Immunodeficiency Virus **(**HIV) diagnosis to treatment initiation, a recent systematic review of studies conducted in Ethiopia before and during the test-and-treat era reported 71% viral load suppression rate, which is considerably lower than the global target of 95%.

- **Comment**: “The abstract is well-structured but somewhat speculative in its conclusion. The claim that higher suppression rates among advanced-disease patients “may reflect more intensive management” should either be toned down or moved to the discussion.”

**Response:** Initially we have toned down the sentence as follows:

Higher suppression rates among patients with advanced disease could partly reflect more intensive care, although this was not directly assessed.

However, the above results changed when the regression analysis was repeated using a binary categorization of nutritional status (undernourished vs. not undernourished), as commented in the Results section. The interaction between nutritional status and adherence was not significant when nutritional status was categorized in this way. Consequently, only the interaction between WHO clinical stage and functional status was included in the multivariable Cox regression model, which was also not statistically significant. Therefore, we concluded the Abstract by stating that *“Lower suppression rates among ambulatory/bedridden patients could partly reflect additional health issues or factors that influence drug response*.”

- Results should consistently present both percentages and absolute numbers (e.g., “290 [70.1%] of 414 clients”).

**Response:** We have revised the entire manuscript accordingly.

**Section: Introduction**

- Change the **Background** to **Introduction** as this section is the summary of the whole introduction.

**Response**: We have revised accordingly.

- The introduction provides a broad overview of HIV epidemiology and treatment goals but could be more concise. There is repetition regarding UNAIDS 90-90-90 and 95-95-95 targets.

**Response:** We have removed the 90-90-90 targets and revised the sentence as follows:

In December 2020, UNAIDS launched the revised 95-95-95 targets to accelerate the progress toward ending AIDS by 2030, with a focus on expanding HIV testing, ART initiation, and viral load suppression (36-40).

- The knowledge gap needs to be sharpened earlier. Ethiopia’s current national viral suppression rates (from DHS, EPHI, or UNAIDS reports) should be explicitly cited to establish context.

**Response:** We have revised the paragraph about Ethiopia as follows:

Ethiopia, one of the world 25 countries with the highest number of new infections, has made efforts to combat the epidemic (41), with 612,925 people living with HIV as of 2021 (42). The country began offering ART in 2003 (43), and by December 2022, 454,257 people were receiving it (44). It introduced “Immediate” ART initiation in 2016 (28, 45-47) and same-day ART initiation in 2017 (43, 48, 49), but there is limited evidence on its effectiveness in routine clinical settings. Ethiopia’s viral load suppression ranged from 91% to 95% according to the Ethiopian Public Health Institute (EPHI) (50, 51) and 96% according to UNAIDS report (52). A recent comparative study from Addis Ababa also reported an overall adolescent viral suppression of 88.3%, with 92.4% among participants in the Operation Triple Zero (OTZ) group and 84.3% in the regular ART group (53). In contrast, a recent systematic review and meta-analysis of studies conducted before and during the test-and treat era reported 71% (54). This discrepancy highlights the need for further assessment of the rate of viral load suppression and its predictors among patients initiating ART in the test-and-treat era.

- Please clarify how this study adds value compared to recent Ethiopian studies (e.g., Belete et al. 2024; Chirnet et al. 2024; Erjino et al. 2023).

**Response**: We added the following text to clarify how this study contributes additional value compared to recent Ethiopian studies.

Unlike earlier Ethiopian studies, the current study focused exclusively on viral load suppression in the test-and-treat era and evaluated the effect of rapid ART initiation on viral load suppression. Furthermore, the study included all patients who newly initiated ART during the study period, regardless of their enhanced adherence counseling (EAC) status.

**Section: Methods**

- The sampling procedure needs clarification. It appears stratified sampling was first used at the facility level followed by simple random sampling within facilities. Please clarify.

**Response**

Dear Reviewer,

We sincerely appreciate your comments, which helped us correct the error in the Abstract section and clarify sampling in the Methods section. In the Methods section, we initially described the sampling technique as a stratified random sampling, but mentioned only simple random sampling in the abstract. We have now revised the description of the sampling technique in both the Abstract and Methods sections to a “proportional stratified random sampling”. We have revised the sampling technique in Methods section as follows:

Patients were selected using proportional stratified random sampling. First, the sample size was proportionally allocated across four health facilities in Shashemene City that provide ART services. Next, a sampling frame of eligible patients was developed for each facility using the ART patient electronic database. Finally, patients were selected through simple random sampling using computer-generated random numbers.

- “Inclusion/exclusion criteria should be stated more explicitly. For example, patients without baseline viral load were excluded, but this is only later noted as a limitation.”

**Response**

We did not exclude patients due to lack of baseline viral load data as most patients did not have this information. Since viral load was not routinely measured for most patients as part of patient care, and we noted this as a limitations to indicate that we could not control for its potential confounding effect. We revised the limitations section to note that viral load was not routinely measured in most patients care. Here’s the revised statement:

Since baseline viral load was not routinely measured in most patients care, we were unable to control for its potential confounding effect, which may have influenced the time to viral suppression.

- The measure of adherence (self-report at first appointment) is weak and prone to bias. This limitation should be acknowledged in the methods as well as in the discussion.

**Response**

***Data Collection* section**

We have added the following text in the Data Collection section:

“Baseline adherence to the treatment was measured by the number of doses missed, as self-report by patients at their first appointment, when they had been on ART for one or two months.”

***Discussion* section:** We have indicated in the revised Discussion section that adherence was assessed through self-report, as follows:

- Baseline adherence to ART, measured by self-report, was also weakly associated with viral load suppression, where patients whose adherence was not good had lower chances of viral load suppression. The lack of strong statistical significance may reflect limitations of self-reported adherence, which can overestimate actual medication-taking behavior. Similar findings have been reported in previous studies, where patients with poor adherence had significantly lower chance of viral suppression (55-57), and those with good adherence had higher chances (58, 59).
- **Comment**: “Ethical clearance is mentioned, but the approval number is not provided – this should be added.”

**Response**: We have added the ethical approval number as follows:

“Ethical clearance was obtained before data collection from the Institutional Research Ethics Review Committee (IRERC) of Madda Walabu University, Shashemene Campus (Ref. No IRERC/014/2023)”.

**Section: Results**

**Comments:**

- **In Survival analysis–based studies** like this one, it is very important to present and interpret both **events** (patients who achieved viral suppression) and **censored cases** (patients who did not achieve suppression during follow-up, were lost to follow-up, transferred out, or died).
- Attrition (loss to follow-up, deaths, transfer-outs) deserves more emphasis, given its potential impact on suppression rates.

**Response**

We have revised the Results section under the heading “Rate of Viral Load Suppression” as follows:

A total of 414 patients were followed-up for a total of 4341.80 person-months. Among these, 99 (79.84%) were censored before the end of the follow-up: 51 (51.52%) due to loss to follow-up or dropout, 28 (28.28%) were transferred out, 7 (7.07%) switched to second-line ART, and 13 (13.13%) died. The remaining 25 (20.16%) censored patients were alive and on ART but had not achieved viral suppression by the end of follow-up. Of those censored before the end of the follow-up, 77(77.78%) were censored before their seventh month on ART.

Among the 414 patients enrolled, 75 of 152 (49.34%; 95% CI: 41.40–57.30) and 235 of 317 (74.13%, 95% CI: 69.31–78.95) patients who were either censored or had available viral load measurements achieved viral suppression at 6 and 12 months of follow-up, respectively, while 290 (70.05%; 95% CI: 65.64–74.46) were virally suppressed at 24 months. The rates of viral suppression were 7.89 (95% CI: 6.29-9.89), 9.51 (95% CI: 8.37-10.80) and 6.68 (95% CI: 5.95-7.49) per 100 person-months at 6, 12 and 24 months of follow up. The median duration to viral suppression was 9.33 months (95% CI: 8.55-10.11) (Figure-1).

Among the 171 patients who initiated ART on the same day as diagnosis, 45 of 67 (67.16%; 95% CI: 55.92–78.41) and 130 of 152 (85.53%; 95% CI: 79.93–91.12) patients who were either censored or had available viral load measurements achieved viral suppression at 6 and 12 months of follow-up, respectively, while 144 (84.21%; 95% CI: 78.75–89.68) were virally suppressed at 24 months.

- **Comment**: The results section is comprehensive, but the narrative repeats information already presented in tables. For clarity, the text should highlight only the most important findings.

**Response:** We have revised accordingly **(**i.e. we have minimized the textual descriptions).

- **Comment**: Interaction terms are briefly mentioned but not well explained. For instance, the reported combined effect of WHO stage and functional status is not clearly presented in the text. Nutritional status*Adherence - specified as Mild*Not good is not clear. Nutritional status is not classified as mild or moderate. You have to specify as undernourished or not.

**Response**

We categorized nutritional status as undernourished versus not undernourished, as indicated in the reviewer’s comment. However, when the regression analysis was repeated using this binary categorization (undernourished vs. not undernourished), the interaction between nutritional status and adherence was not significant in the model that contains only nutritional status, adherence and their interaction term (Table 1).

Table 1 Cox regression of nutritional status, adherence and their interaction term

Therefore, only the interaction between WHO clinical stage and functional status was included in the multivariable Cox regression model, although this interaction was not statistically significant in multivariable Cox regression (Table 2), despite its biological plausibility.

Table 2 Multivariable Cox regression of candidate predictors with interaction term of baseline WHO clinical stage and functional status

When comparing the Global test results of multiple Cox regression models with and without the interaction term, the model without the interaction term showed non-significant p-values for all variables (Table 3), whereas the model with the interaction term showed significance for rapid ART initiation (p-value = 0.0468) (Table 4). The overall Global test p-value was also less significant in the model without the interaction term (p-value = 0.4547) compared to the model with the interaction term (p-value = 0.3110).

Based on these results, we preferred the model without the interaction term and have accordingly revised the textual description of the results and the multivariable analysis table. The Abstract and Discussion sections have also been updated to reflect these changes.

Table 4 Test of proportional-hazards assumption

Table 3 Test of proportional-hazards assumption

- **Comment:** Rates of suppression (incidence rates vs. cumulative proportions) are sometimes difficult to follow. A clearer distinction between these metrics is recommended.

**Response:** The textual description has been revised to explicitly indicate that the 70.05% refers to the cumulative incidence. The incidence rate remains presented as the number of events per 100 person-months, as reported previously.

“A total of 414 patients were followed up for a total of 4341.80 months. Among them, 290 achieved viral suppression, corresponding to a cumulative incidence of 70.05% (95% CI: 65.64–74.46). The remaining 124 (29.95%) were censored at the end of the follow-up period.”

- **Comments**:
- “Best practice
- You need to Present **KM curves** only for variables of **primary interest** (the study’s main predictors, significant or nearly significant ones).
- For non-significant predictors, a good compromise is:
  - Mention in the methods/results that KM curves were examined for all candidate predictors, but only significant (or key clinical) ones are shown. But in your report especially in the final model, most of the variables were not even candidate for the Multivariable Cox-regression.”

**Response**:

We sincerely appreciate your guidance on how to present KM curves of evaluated variables. We have revised the manuscript mentioning in the Methods section as you recommended, “KM curves were examined for all candidate predictors, but only significant ones are shown”. In the revised manuscript, we have cited the KM curves only for variables of **primary interest** (the study’s main predictors, significant or nearly significant ones). Therefore, we presented KM curve for the overall viral suppression, Rapid ART initiation, nutritional status, functional status and ART adherence.

**Section: Discussion**

- Comment: The discussion contextualizes findings against regional and global studies but is sometimes speculative. The statement about advanced-disease patients achieving faster suppression due to “more intensive care” should be tempered unless supported by references.

**Response:** We have reduced the speculative feature of the discussion by replacing phrases such as “may have influenced”, “may be due to”, “may indicate”, etc. with “may partly be attributed”, “may suggest that”, “possibly due to”, “may be partly explained by”, “could reflect”, and “possibly because”.

The finding “advanced-disease patients achieving faster suppression” is changed as the WHO clinical stage III/IV is only marginally significant in the revised model. Therefore, the phrase “more intensive care” is deleted from the discussion.

- **Comment**: Comparative analysis with Ethiopia-specific studies could be expanded, highlighting differences in study setting, design, or population.

**Response:** We have expanded the comparative analysis with Ethiopia-specific studies and highlighted the differences in study settings, design, or population characteristics.

- **Comments**: The policy implications should be stronger and more actionable for example:
  - Prioritizing same-day ART initiation through strengthened counseling and system support.
  - Expanding nutritional support not only for moderate/severe malnutrition but also for mild malnutrition.
  - Enhancing adherence support for patients at earlier stages of disease who may otherwise delay viral suppression.

**Response**: These are really strong and very relevant recommendations. Hence, we have revised the Conclusion section as follows:

Despite the progress made, our findings highlight the need for more targeted interventions. Same-day ART initiation should be prioritized through strengthened counseling and system support. Patients who delay ART initiation require closer follow-up to prevent treatment delays. Nutritional support should be expanded not only for those with severe malnutrition but any mildly undernourished patients. Furthermore, adherence support should be enhanced for all patients, particularly those with limited functional status, regardless of WHO clinical stage, as they may otherwise experience delayed viral suppression.

Ultimately, the path to viral suppression is not the same for every patient. But by understanding the factors that influence this journey, we can take meaningful steps to close the gap and move closer to the global 95% target, improving both individual and public health outcomes. We recommend future prospective studies that control for baseline viral load, provide more precise estimates of the time to viral load suppression, and include objective measures of treatment adherence.

- **Comment**: The limitations section is appropriate but could mention missing baseline viral load data more prominently, as this is a critical factor influencing suppression dynamics.

**Response**: We have revised the limitations follows:

Despite these insights, our study has some limitations. Because baseline viral load was not routinely measured in most patients care, we were unable to account for its potential influence on the time to viral suppression. Prior studies (54, 58-60) have shown that baseline viral load was significantly associated with time to viral load suppression. Additionally, adherence was assessed through self-reported missed doses, which may have led to an overestimation of good adherence due to social desirability bias.

**Minor Comments**

1. Ensure consistency in reporting percentages (e.g., sometimes one decimal place, sometimes two).

**Response**: We have revised accordingly; we used two decimal places for all percentages except for p-values, where we used three decimal places.

2. In tables, consider reordering variables for readability (e.g., functional status and WHO stage together).

**Response**: We have revised accordingly in both tables.

3. Improve grammar/phrasing in some areas, e.g., “clients whose viral load was determined” → “clients with available viral load measurements.”

**Response**: We have revised as per the constructive comment.

4. Figures and tables should be self-explanatory; table captions could be expanded slightly to clarify denominators.

**Response:** We have revised as per the constructive comments.

5. Consider condensing overly detailed background statistics (e.g., global HIV numbers) to focus more on Ethiopia.

**Response**: We have revised the Background section replacing most of *global HIV numbers* by Ethiopian HIV statistics.

**References**

1.	Huang YC, Sun HY, Chuang YC, Huang YS, Lin KY, Huang SH, et al. Short-term outcomes of rapid initiation of antiretroviral therapy among HIV-positive patients: real-world experience from a single-centre retrospective cohort in Taiwan. BMJ Open. 2019;9(9). doi: 10.1136/bmjopen-2019-033246.

2.	Grinsztejn B, Hosseinipour MC, Ribaudo HJ, Swindells S, Eron J, Chen YQ, et al. Effects of early versus delayed initiation of antiretroviral treatment on clinical outcomes of HIV-1 infection: results from the phase 3 HPTN 052 randomised controlled trial. Lancet Infect Dis. 2014;14(4):281-90. Epub 20140304. doi: 10.1016/s1473-3099(13)70692-3. PubMed PMID: 24602844; PubMed Central PMCID: PMC4144040.

3.	Lundgren JD, Babiker AG, Gordin F, Emery S, Grund B, Sharma S, et al. Initiation of Antiretroviral Therapy in Early Asymptomatic HIV Infection. N Engl J Med. 2015;373(9):795-807. Epub 20150720. doi: 10.1056/NEJMoa1506816. PubMed PMID: 26192873; PubMed Central PMCID: PMC4569751.

4.	Pilcher CD, Ospina-Norvell C, Dasgupta A, Jones D, Hartogensis W, Torres S, et al. The Effect of Same-Day Observed Initiation of Antiretroviral Therapy on HIV Viral Load and Treatment Outcomes in a US Public Health Setting. J Acquir Immune Defic Syndr. 2017;74(1):44-51. doi: 10.1097/qai.0000000000001134. PubMed PMID: 27434707; PubMed Central PMCID: PMC5140684.

5.	Mateo-Urdiales A, Johnson S, Smith R, Nachega JB, Eshun-Wilson I. Rapid initiation of antiretroviral therapy for people living with HIV. Cochrane Database Syst Rev. 2019;6(6). doi: 10.1002/14651858.CD012962.pub2.

6.	Brown LB, Havlir DV, Ayieko J, Mwangwa F, Owaraganise A, Kwarisiima D, et al. High levels of retention in care with streamlined care and universal test and treat in East Africa. AIDS. 2017;30(18). doi: 10.1097/QAD.0000000000001250.

7.	Khan S, Spiegelman D, Walsh F, Mazibuko S, Pasipamire M, Chai B, et al. Early access to antiretroviral therapy versus standard of care among HIV-positive participants in Eswatini in the public health sector: the MaxART stepped-wedge randomized controlled trial. J Int AIDS Soc2020.

8.	Koenig SP, Dorvil N, Dévieux JG, Hedt-Gauthier BL, Riviere C, Faustin M, et al. Same-day HIV testing with initiation of antiretroviral therapy versus standard care for persons living with HIV: A randomized unblinded trial. PLOS Medicine. 2017;14(7):e1002357. doi: 10.1371/journal.pmed.1002357.

9.	Mody A, Sikazwe I, Namwase AS, Wa Mwanza M, Savory T, Mwila A, et al. Effects of implementing universal and rapid HIV treatment on initiation of antiretroviral therapy and retention in care in Zambia: a natural experiment using regression discontinuity. Lancet HIV. 2021;8(12):e755-e65. Epub 20211014. doi: 10.1016/s2352-3018(21)00186-7. PubMed PMID: 34656208; PubMed Central PMCID: PMC8639712.

10.	Mugenyi L, Nanfuka M, Byawaka J, Agaba C, Mijumbi A, Kagimu D, et al. Effect of universal test and treat on retention and mortality among people living with HIV-infection in Uganda: An interrupted time series analysis. PLOS ONE. 2022;17. doi: 10.1371/journal.pone.0268226.

11.	Havlir D, Lockman S, Ayles H, Larmarange J, Chamie G, Gaolathe T, et al. What do the Universal Test and Treat trials tell us about the path to HIV epidemic control? J Int AIDS Soc. 2020;23. doi: 10.1002/jia2.25455.

12.	Labhardt ND, Ringera I, Lejone TI, Klimkait T, Muhairwe J, Amstutz A, et al. Effect of Offering Same-Day ART vs Usual Health Facility Referral During Home-Based HIV Testing on Linkage to Care and Viral Suppression Among Adults With HIV in Lesotho: The CASCADE Randomized Clinical Trial. JAMA. 2018;319(11):1103-12. doi: 10.1001/jama.2018.1818.

13.	Lebelonyane R, Bachanas P, Block L, Ussery F, Abrams W, Roland M, et al. Rapid antiretroviral therapy initiation in the Botswana Combination Prevention Project: a quasi-experimental before and after study. Lancet HIV. 2020;7(8). doi: 10.1016/S2352-3018(20)30187-9.

14.	Danel C, Moh R, Gabillard D, Badje A, Le Carrou J, Ouassa T, et al. A Trial of Early Antiretrovirals and Isoniazid Preventive Therapy in Africa. N Engl J Med. 2015;373(9):808-22. Epub 20150720. doi: 10.1056/NEJMoa1507198. PubMed PMID: 26193126.

15.	Hayes RJ, Donnell D, Floyd S, Mandla N, Bwalya J, Sabapathy K, et al. Effect of Universal Testing and Treatment on HIV Incidence - HPTN 071 (PopART). N Engl J Med. 2019;381(3):207-18. doi: 10.1056/NEJMoa1814556. PubMed PMID: 31314965; PubMed Central PMCID: PMC6587177.

16.	Baisley K, Orne-Gliemann J, Larmarange J, Plazy M, Collier D, Dreyer J, et al. Early HIV treatment and survival over six years of observation in the ANRS 12249 Treatment as Prevention Trial. HIV Med. 2022;23(8):922-8. Epub 20220226. doi: 10.1111/hiv.13263. PubMed PMID: 35218300; PubMed Central PMCID: PMC9545558.

17.	Stevens WS, Gous NM, MacLeod WB, Long LC, Variava E, Martinson NA, et al. Multidisciplinary Point-of-Care Testing in South African Primary Health Care Clinics Accelerates HIV ART Initiation but Does Not Alter Retention in Care. J Acquir Immune Defic Syndr. 2017;76(1):65-73. doi: 10.1097/qai.0000000000001456. PubMed PMID: 28542080.

18.	Rosen S, Maskew M, Larson BA, Brennan AT, Tsikhutsu I, Fox MP, et al. Simplified clinical algorithm for identifying patients eligible for same-day HIV treatment initiation (SLATE): Results from an individually randomized trial in South Africa and Kenya. PLoS Med. 2019;16(9). doi: 10.1371/journal.pmed.1002912.

19.	Alhaj M, Amberbir A, Singogo E, Banda V, van Lettow M, Matengeni A, et al. Retention on antiretroviral therapy during Universal Test and Treat implementation in Zomba district, Malawi: a retrospective cohort study. J Int AIDS Soc. 2019;22(2):e25239. doi: 10.1002/jia2.25239. PubMed PMID: 30734510; PubMed Central PMCID: PMC6367572.

20.	Hamooya BM, Mutembo S, Muyunda B, Mweebo K, Kancheya N, Sikazwe L, et al. HIV test-and-treat policy improves clinical outcomes in Zambian adults from Southern Province: A multicenter retrospective cohort study. Frontiers in Public Health. 2023;11. doi: 10.3389/fpubh.2023.1244125.

21.	Chauke P, Huma M, Madiba S. Lost to follow up rate in the first year of ART in adults initiated in a universal test and treat programme: a retrospective cohort study in Ekurhuleni District, South Africa. Pan Afr Med J. 2020;37. doi: 10.11604/pamj.2020.37.198.25294.

22.	Hirasen K, Fox MP, Hendrickson CJ, Sineke T, Onoya D. HIV Treatment Outcomes Among Patients Initiated on Antiretroviral Therapy Pre and Post-Universal Test and Treat Guidelines in South Africa. Ther Clin Risk Manag. 2020;16:169-80. Epub 20200304. doi: 10.2147/tcrm.S227290. PubMed PMID: 32184609; PubMed Central PMCID: PMC7061415.

23.	Kimanga DO, Oramisi VA, Hassan AS, Mugambi MK, Miruka FO, Muthoka KJ, et al. Uptake and effect of universal test-and-treat on twelve months retention and initial virologic suppression in routine HIV program in Kenya. PLOS ONE. 2022;17(11):e0277675. doi: 10.1371/journal.pone.0277675.

24.	Lilian RR, Rees K, McIntyre JA, Struthers HE, Peters RPH. Same-day antiretroviral therapy initiation for HIV-infected adults in South Africa: Analysis of routine data. PLOS ONE. 2020;15(1):e0227572. doi: 10.1371/journal.pone.0227572.

25.	Nicol E, Basera W, Mukumbang FC, Cheyip M, Mthethwa S, Lombard C, et al. Linkage to HIV Care and Early Retention in Care Rates in the Universal Test-and-Treat Era: A Population-based Prospective Study in KwaZulu-Natal, South Africa. AIDS Behav. 2022;27(4). doi: 10.1007/s10461-022-03844-w.

26.	Rosen S, Maskew M, Fox MP, Nyoni C, Mongwenyana C, Malete G, et al. Initiating Antiretroviral Therapy for HIV at a Patient’s First Clinic Visit: The RapIT Randomized Controlled Trial. PLOS Medicine. 2016;13(5):e1002015. doi: 10.1371/journal.pmed.1002015.

27.	Stafford KA, Odafe SF, Lo J, Ibrahim R, Ehoche A, Niyang M, et al. Evaluation of the clinical outcomes of the Test and Treat strategy to implement Treat All in Nigeria: Results from the Nigeria Multi-Center ART Study. PLoS One. 2019;14(7). doi: 10.1371/journal.pone.0218555.

28.	Ahmed I, Demissie M, Worku A, Gugsa S, Berhane Y. Effectiveness of same-day antiretroviral therapy initiation in retention outcomes among people living with human immunodeficiency virus in Ethiopia: empirical evidence. BMC Public Health. 2020;20:1-11.

29.	Beyene H. long-term Retention on antiretroviral therapy during universal test and treat implementation in Addis Ababa, Ethiopia. International Institute for Primary Health Care-Ethiopia (IPHCe): Addis Ababa; 2024.

30.	Bantie B, Seid A, Kerebeh G, Alebel A, Dessie G. Loss to follow-up in "test and treat era" and its predictors among HIV-positive adults receiving ART in Northwest Ethiopia: Institution-based cohort study. Front Public Health. 2022;10. doi: 10.3389/fpubh.2022.876430.

31.	Ahmed I, Demissie M, Worku A, Gugsa S, Berhane Y. Adherence to antiretroviral treatment among people who started treatment on the same-day of HIV diagnosis in Ethiopia: A multicenter observational study. HIV/AIDS-Research and Palliative Care. 2021;13:983-91. doi: 10.2147/HIV.S337073.

32.	Ahmed I, Demissie M, Worku A, Gugsa S, Berhane Y. Virologic outcomes of people living with human immunodeficiency virus who started antiretroviral treatment on the same-day of diagnosis in Ethiopia: a multicenter observational study. PLoS One. 2021;16(9):e0257059.

33.	Girum T, Yasin F, Dessu S, Zeleke B, Geremew M. “Universal test and treat” program reduced TB incidence by 75% among a cohort of adults taking antiretroviral therapy (ART) in Gurage zone, South Ethiopia. Tropical diseases, travel medicine and vaccines. 2020;6:1-7.

34.	Girum T, Yasin F, Wasie A, Shumbej T, Bekele F, Zeleke B. The effect of “universal test and treat” program on HIV treatment outcomes and patient survival among a cohort of adults taking antiretroviral treatment (ART) in low income settings of Gurage zone, South Ethiopia. AIDS Research and Therapy. 2020;17(1):19. doi: 10.1186/s12981-020-00274-3.

35.	Tesfaye B, Ermias D, Moges S, Astatkie A. Effect of the Test and Treat Strategy on Mortality Among HIV-Positive Adult Clients on Antiretroviral Treatment in Public Hospitals of Addis Ababa, Ethiopia. HIV AIDS (Auckl). 2021;13:349–60. doi: 10.2147/HIV.S303557.

36.	Kemnic TR, Gulick PG. HIV Antiretroviral Therapy. StatPearls. Treasure Island (FL): StatPearls Publishing Copyright © 2025, StatPearls Publishing LLC.; 2025.

37.	World Health Organization [WHO]. Global progress report on HIV, viral hepatitis and sexually transmitted infections, 2021. Accountability for the global health sector strategies 2016–2021: actions for impact. Geneva: World Health Organization, 2023 Contract No.: CC BY-NC-SA 3.0 IGO.

38.	The Joint United Nations Programme on HIV/AIDS [UNAIDS]. Confronting inequalities: Lessons for pandemic responsesfrom 40 years of AIDS. Geneva: Global AIDS Update 2021, 2021.

39.	The Joint United Nations Programme on HIV/AIDS [UNAIDS]. In Danger: UNAIDS Global AIDS Update 2022. 2022 [October 10, 2022]. Available from: <https://www.unaids.org/en/resources/documents/2022/in-danger-global-aids-update>.

40.	Frescura L, Godfrey-Faussett P, Feizzadeh A, El-Sadr W, Syarif O, Ghys PD, et al. Achieving the 95 95 95 targets for all: A pathway to ending AIDS. PLOS ONE. 2022;17(8):e0272405. doi: 10.1371/journal.pone.0272405.

41.	Federal HIV/AIDS Prevention and Control Office [FHAPCO]. HIV Prevention in Ethiopia: National Road Map 2018 - 2020 2018. Available from: <https://ethiopia.unfpa.org/sites/default/files/pub-pdf/HIV%20Prevention%20in%20Ethiopia%20National%20Road%20Map%202018%20-%202020%20FINAL_FINAL.pdf>.

42.	Ethiopian Public Helth Institute [EPHI]. HIV Related Estimates and Projection in Ethiopia. 2022.

43.	Federal Minister of Health, Ethiopia [FMOH]. National Consolidated Guidelines for Comprehensive HIV Prevention, Care and Treatment 2018. Available from: <https://www.afro.who.int/sites/default/files/2019-04/National%20Comprehensive%20HIV%20Care%20%20Guideline%202018.pdf>.

44.	Mantell JE, Zech JM, Masvawure TB, Assefa T, Molla M, Block L, et al. Implementing six multi-month dispensing of antiretroviral therapy in Ethiopia: perspectives of clients and healthcare workers. BMC Health Services Research. 2023;23(1):563. doi: 10.1186/s12913-023-09549-7.

45.	Girum T, Yasin F, Wasie A, Shumbej T, Bekele F, Zeleke B. The effect of “universal test and treat” program on HIV treatment outcomes and patient survival among a cohort of adults taking antiretroviral treatment (ART) in low income settings of Gurage zone, South Ethiopia. AIDS Research and Therapy. 2020;17:1-9.

46.	Bantie B, Kassaw Yirga G, Abate MW, Amare AT, Nigat AB, Tigabu A, et al. Delayed ART initiation in “Test and Treat era” and its associated factors among adults receiving antiretroviral therapy at public health institutions in Northwest Ethiopia: A multicenter cross-sectional study. PLoS One. 2022;17(7):e0271127.

47.	Bayisa L, Abera T, Mulisa D, Mosisa G, Mosisa A, Tolosa T, et al. Time to Antiretroviral Therapy Initiation and Its Predictors Among Newly Diagnosed HIV-Positive People in Nekemte Town, Western Ethiopia: Claim of Universal Test and Treat. HIV/AIDS-Research and Palliative Care. 2021:959-72.

48.	World Health Organization [WHO]. Guidelines for managing advanced HIV disease and rapid initiation of antiretroviral therapy 2017. Available from: <https://iris.who.int/bitstream/handle/10665/255884/9789241550062-eng.pdf?sequence=1>.

49.	Moges NA, Adesina OA, Okunlola MA, Berhane Y. Barriers and facilitators of same-day antiretroviral therapy initiation among people newly diagnosed with HIV in Ethiopia: qualitative study using the transtheoretical model of behavioral change. JMDH. 2020:1801-15.

50.	Ministry of Health - Ethiopia (MOH-E). National Guidelines for Comprehensive HIV Prevention, Care and Treatment 2022.

51.	Ministry of Health - Ethiopia (MOH-E). National Comprehensive HIV Prevention, Care and Treatment Training Participant Manual 2023.

52.	UNAIDS. Impact of recent U.S. shifts on the global HIV response 2025. Available from: <https://www.unaids.org/en/resources/presscentre/featurestories/2025/february/20250213_ethiopia>.

53.	Tafere GW, Hunduma F, Yesuf A. Viral suppression rate at operation triple zero (Otz) and regular art follow-Up programs and associated factors among adolescent clients of Addis Ababa Ethiopia: a comparative cross-sectional study. Virol J. 2023;20:208. doi: 10.1186/s12985-023-02176-y.

54.	Melak D, Bayou FD, Yasin H, Zerga AA, Wagaye B, Ayele FY, et al. Virological Suppression and its Predictors Among HIV/AIDS Patients on Antiretroviral Therapy in Ethiopia: Systematic Review and Meta-analysis. Open Forum Infect Dis. 2024;11(4). doi: 10.1093/ofid/ofae168.

55.	Chirnet AT, Habtewold EM, Aman H, Wakwoya EB, Workie SG. Time to viral load suppression and its predictors among people living with HIV on antiretroviral therapy in Gebi Resu zone, Afar Region, Ethiopia, 2023. Front Public Health. 2024;12:1384787. Epub 20241028. doi: 10.3389/fpubh.2024.1384787. PubMed PMID: 39529713; PubMed Central PMCID: PMC11550932.

56.	Anito AA, Lenjebo TL, Woticha E, Solomon F. Magnitude of viral load suppression and associated factors among clients on antiretroviral therapy in public hospitals of Hawassa City Administration, Ethiopia. HIV/AIDS (Auckland, NZ). 2022;14:529.

57.	Waju B, Dube L, Ahmed M, Assefa SS. Unsuppressed Viral Load Level in Public Health Facilities: Nonvirological Predictors among Adult Antiretroviral Therapy Users in Southwestern Ethiopia. HIV AIDS (Auckl). 2021;13:513-26. Epub 20210514. doi: 10.2147/HIV.S304653. PubMed PMID: 34017201; PubMed Central PMCID: PMC8131002.

58.	Muhie NS. Predictors for viral load suppression among HIV positive adults under ART treatment in University of Gondar Comprehensive Specialized Hospital: retrospective cohort study. Scientific Reports. 2024;14(1):3258. doi: 10.1038/s41598-024-53569-0.

59.	Hussen S, Mama M, Mekonnen B, Yihune a, Shegaze M, Boti N, et al. Predictors of Time to Viral Load Suppression of Adult PLWHIV on ART in Arba Minch General Hospital: A Follow up Study. Ethiopian Journal of Health Sciences. 2019;29(6). doi: 10.4314/ejhs.v29i6.12.

60.	Belete MB, Bitew A, Mulatu K. Viral load suppression and its predictor among HIV seropositive people who receive enhanced adherence counseling at public health institutions in Bahir Dar, Northwest Ethiopia. Retrospective follow-up study. PLoS One. 2024;19(5):e0303243. Epub 20240513. doi: 10.1371/journal.pone.0303243. PubMed PMID: 38739646; PubMed Central PMCID: PMC11090359.
