# Supplementary material for: Viral suppression among adolescents and adults living with human immunodeficiency virus in Ethiopia during the test-and-treat era
Source: PLoS One. 2026 Nov 11;21(11):e0362375. doi: 10.1371/journal.pone.0362375 (PMC13754153; doi:10.1371/journal.pone.0362375)
Supplement: Supplementary file 7 [file pone.0362375.s010.docx]

**Response to Academic Editor and Reviewer**

**Manuscript Title**: Viral suppression among adolescents and adults living with Human Immunodeficiency Virus in Ethiopia during the test -and-test era

Manuscript ID: PONE-D-25-07030R1

Dear Academic Editor and Reviewer,

We would like to express our sincere appreciation to you for your valuable time, insightful comments, and constructive suggestions, which have greatly improved the quality and clarity of this manuscript. Your thoughtful feedback has helped refine our work and strengthen the interpretation of our findings. We are deeply grateful for your professional contribution to enhancing the overall scientific rigor of this work. We have responded to the comments and concerns as follows:

**Additional Editor Comments**

**Comment**: In table 3, all variables should have values. For example, rapid ART initiation, Yes=A; No=B

**Response**: We appreciate the editor’s comment. We have included the number of events and censored cases for each category of variables in the multiple regression. However, as the table became very congested, we deleted the column showing p-values for the adjusted regression. The significance and precision of the estimates can be understood from confidence interval.

**Comment**: Figures 3-5 were well cited. But the concept for each respective figure was too short.

**Response**: We appreciate the editor’s comment. We have revised the manuscript by further elaborating the concepts as follows:

- Patients who were undernourished at baseline had a significantly lower rate of achieving viral load suppression compared with patients who were not undernourished. Specifically, patients who were undernourished at baseline had a 46% lower chance of earlier viral load suppression (AHR = 0.54; 95% CI: 0.36–0.81). The Kaplan-Meier survival curves demonstrated a clear separation between the two groups, with undernourished patients experiencing delayed viral load suppression throughout the follow-up period (**Fig 3**).
- Patients who had an ambulatory or bedridden functional status at baseline had an 87% lower chance of earlier achieving viral load suppression than those with a working functional status (AHR = 0.13; 95% CI: 0.07–0.27). The Kaplan-Meier survival curves demonstrated a marked difference in time to viral load suppression between the two groups, with ambulatory/bedridden patients experiencing substantially delayed viral load suppression throughout the follow-up period (**Fig 4**).
- Patients with not good baseline ART adherence had a 36% lower chance of achieving earlier viral load suppression compared with patients who had good ART adherence at baseline (AHR = 0.64; 95% CI: 0.41–0.99). The Kaplan-Meier survival curves demonstrated that patients with not good adherence experienced delayed viral load suppression throughout the follow-up period compared with those who maintained good adherence (**Fig 5**).

**Comment**

- Line 112: **Materials and Methods**
- Line 113: **Study area and setting**

**Response**: We appreciate the editor’s comments. We have revised the manuscript accordingly.

**Comment**

- Add the p-value to the description part

**Response**: We appreciate the editor’s comments. We have revised the manuscript by including the p-value as follows:

Example: (AHR= 2.05; 95% CI = 1.58–2.67; p <0.001).

**Comment**

- The list of abbreviations (no need for two columns) and the declaration section should be above the list of references.

**Response**: We appreciate the editor’s comments. We have revised the manuscript accordingly.

**Comment**

- The reference list should be written based on the standard format.

**Response**: We appreciate the editor’s comments. We have revised the in-text reference citations using square brackets and reformatted the reference list according to the journal’s standard reference format.

**Comment**

- Upload figures in a separate file as TIF/or TIFF version without their captions, captions remain within the main manuscript where cited and at list of ‘supporting’ information.

**Response**: We appreciate the editor’s comments. We have uploaded the figures in a separate file as TIF version without their captions. We kept the captions within the main manuscript where figures are cited and at list of ‘supporting’ information.

**Comment**

- What is your basis for citing the ‘supporting information’ in the ‘Data analysis’ section?

**Response**: Thank you for raising this point. We cited the ‘supporting information’ because it contains the detailed statistical diagnostics and model-comparison results underlying the analytical decisions described in the Data Analysis section. Specifically, S1 Table presents the VIF results used to assess multicollinearity; S2 and S3 Tables provide the global test results for the Cox regression models with and without the interaction term, respectively; and S1 Fig presents the Cox-Snell residual plot used to assess model fit. These materials provide transparency and allow readers to verify the statistical procedures and model-selection decisions without overloading the main manuscript with detailed diagnostic results.

**Reviewer Comments**

**Comment #1**

The introduction of the abstract should be 1–2 sentences that state the public health problem, clinical gap, or knowledge gap your study addresses. Stating the systematic review in the introduction in not addressing the gap. Please revise.

**Response:** We appreciate the reviewer’s comments and revised the section as follows:

“Despite progress in reducing the time from Human Immunodeficiency Virus **(**HIV) diagnosis to treatment initiation, achieving sustained viral load suppression remains a major public health challenge among people living with HIV in Ethiopia. However, evidence on viral load suppression and its predictors among adolescents and adults during the test-and-treat era remains limited.”

**Comment #2.**

Please clearly indicate what time point is the suppression rate is measured in objective and in the results of the abstract. This is also valid for the main article where follow up time is referred as time point.

**Response**: We appreciate the reviewer’s comments and revised the manuscript accordingly.

**Comment #3**

In the methods of abstract it says the patients were followed up for 2 years. But in the text it says patients starting ART until Dec 2021 were included and followed up until Dec 2022. So that means there is less than 2 years of follow up for some.

**Response**: We appreciate the reviewer’s comments. Our study was open cohort and patients were enrolled from January 2018 to Dec 2021 and followed-up until December 2022. Therefore, 2 years is the maximum follow-up period for patients who enrolled before Dec 2020 and who did not develop outcome of interest, die, loss to follow-up, or transfer-out. We have updated the manuscript by revising the sentence as follows:

“Patients were selected using a proportional stratified random sampling technique and followed for a maximum of two years in public health facilities in Shashemene City.”

**Comment #4**

The Anderson methods is described in the introduction. It should be in methods.

**Response**: We appreciate the reviewer’s comments. We added Anderson method in the Background section in response to the previous reviewer’s comment on the Background section as follows:

**Comment #5**

Could you clarify if the censored cases includes those who did not achieve the viral suppression? If yes please revise the definition.

**Response**: We appreciate the reviewer’s comments. We have updated the manuscript by revising the operational definition as follows:

“**Censored**: lost to follow-up or transferred out or switched to second-line or died during follow-up period between January 2018 and December 2022 or alive and on ART but did not achieve viral suppression at the end of follow-up (December 2022).”

**Comment #6**

The result section of the viral suppression rate is little confusing. For better visualization, I suggest using a flowchart so we can easily follow up the numbers. And present clearly the suppression in terms of time point.

**Response**: We appreciate the reviewer’s comments. We have updated the manuscript by adding the following flow diagram.

A total of 414 patients were followed-up between January 2018 and December 2022

At the end of 6 months of follow-up, 152 were either censored or had available viral load measurements (Total person–months = 950.47)

- 75 were suppressed
- 43 were LTFU/dropout
- 28 were transfer-out
- 6 died

At the end of 12 months of follow-up, 317 were either censored or had available viral load measurements (Total person–months = 2472.03)

- 235 were suppressed
- 47 were LTFU/dropout
- 28 were transfer-out
- 7 died

- 290 were suppressed
- 25 were alive and on ART but had not yet achieved viral suppression
- 51 were LTFU/dropout
- 28 were transfer-out
- 7 Switched to 2^nd^ line ART
- 13 died

At the end of 24 months of follow-up, 414 were either censored or had available viral load measurements (Total person–months = 4341.80)

Figure-1. Flow diagram of follow-up and viral suppression among patients on antiretroviral treatment at public health facilities in Shashemene City, Ethiopia, Jan 2018–Dec 2022

**Comment #7:**

The discussion indeed is more rich but could be better written. The section where the comparisation is made with others studies that have same results could be more summarised and easy to read. If there is specific characteristic of the study it could be emphasised. Again what additional knowledge this study is brought should be emphasised.

**Response**: We appreciate the reviewer’s comments. We have summarized the section where the comparison is made with other studies that have same results.

“The proportion of viral suppression in our study was consistent with findings from previous Ethiopian studies conducted during the test-and-treat era (65). It was also comparable to the pooled estimate reported in a recent systematic review of studies conducted in Ethiopia among different populations and using different ART regimens (59). Similarly, the pooled estimate from five studies among adults receiving first-line ART during the test-and-treat era was consistent with our findings (66-70). Among same-day ART initiators, the proportion of viral suppression in our study was also comparable to findings from previous Ethiopian studies, including a cross-sectional study assessing suppression at 12 months (56) and a retrospective cohort study (48).

Our findings therefore complement previous Ethiopian evidence by providing longitudinal data on viral suppression over the first two years of treatment among patients who initiated ART exclusively during the test-and-treat era. Unlike studies that specifically focused on same-day ART initiators, our study included patients regardless of the interval between HIV diagnosis and ART initiation, thereby providing a broader assessment of viral suppression among patients receiving ART in routine care.”

Best regards,

Habtemu Jarso Hebo (Corresponding author)
