# Supplementary material for: Viral suppression among adolescents and adults living with human immunodeficiency virus in Ethiopia during the test-and-treat era
Source: PLoS One. 2026 Nov 11;21(11):e0362375. doi: 10.1371/journal.pone.0362375 (PMC13754153; doi:10.1371/journal.pone.0362375)
Supplement: Peer review history

# Peer review: Decision Letter 0

**DOI:** 10.1371/journal.pone.0362375.r001

**Authors:** Mengistu Zenebe

21 Aug 2025

Please submit your revised manuscript by Oct 05 2025 11:59PM. If you will need more time than this to complete your revisions, please reply to this message or contact the journal office at plosone@plos.org. When you're ready to submit your revision, log on to https://www.editorialmanager.com/pone/ and select the 'Submissions Needing Revision' folder to locate your manuscript file.

- A rebuttal letter that responds to each point raised by the academic editor and reviewer(s). You should upload this letter as a separate file labeled 'Response to Reviewers'.
- A marked-up copy of your manuscript that highlights changes made to the original version. You should upload this as a separate file labeled 'Revised Manuscript with Track Changes'.
- An unmarked version of your revised paper without tracked changes. You should upload this as a separate file labeled 'Manuscript'.

If applicable, we recommend that you deposit your laboratory protocols in protocols.io to enhance the reproducibility of your results. Protocols.io assigns your protocol its own identifier (DOI) so that it can be cited independently in the future. For instructions see: https://journals.plos.org/plosone/s/submission-guidelines#loc-laboratory-protocols. Additionally, PLOS ONE offers an option for publishing peer-reviewed Lab Protocol articles, which describe protocols hosted on protocols.io. Read more information on sharing protocols at https://plos.org/protocols?utm_medium=editorial-email&utm_source=authorletters&utm_campaign=protocols.

We look forward to receiving your revised manuscript.

Kind regards,

Mengistu Hailemariam Zenebe, PhD

Academic Editor

PLOS ONE

Journal Requirements:

When submitting your revision, we need you to address these additional requirements.

1. Please ensure that your manuscript meets PLOS ONE's style requirements, including those for file naming. The PLOS ONE style templates can be found at

https://journals.plos.org/plosone/s/file?id=wjVg/PLOSOne_formatting_sample_main_body.pdf and

https://journals.plos.org/plosone/s/file?id=ba62/PLOSOne_formatting_sample_title_authors_affiliations.pdf

2. Please include your full ethics statement in the ‘Methods’ section of your manuscript file. In your statement, please include the full name of the IRB or ethics committee who approved or waived your study, as well as whether or not you obtained informed written or verbal consent. If consent was waived for your study, please include this information in your statement as well.

3. If the reviewer comments include a recommendation to cite specific previously published works, please review and evaluate these publications to determine whether they are relevant and should be cited. There is no requirement to cite these works unless the editor has indicated otherwise.

4. Please review your reference list to ensure that it is complete and correct. If you have cited papers that have been retracted, please include the rationale for doing so in the manuscript text, or remove these references and replace them with relevant current references. Any changes to the reference list should be mentioned in the rebuttal letter that accompanies your revised manuscript. If you need to cite a retracted article, indicate the article’s retracted status in the References list and also include a citation and full reference for the retraction notice.

[Note: HTML markup is below. Please do not edit.]

Reviewers' comments:

Reviewer's Responses to Questions

**Comments to the Author**

1. Is the manuscript technically sound, and do the data support the conclusions?

Reviewer #1: No

Reviewer #2: Yes

**********

2. Has the statistical analysis been performed appropriately and rigorously?

Reviewer #1: Yes

Reviewer #2: Yes

**********

3. Have the authors made all data underlying the findings in their manuscript fully available?

The PLOS Data policy

Reviewer #1: Yes

Reviewer #2: Yes

**********

4. Is the manuscript presented in an intelligible fashion and written in standard English?

Reviewer #1: Yes

Reviewer #2: No

**********

Reviewer #1: Abstract:

Well-presented, which gave readers background of the problem, the aim, sampling technique

, type of analysis, the results and the contribution were well indicated. Suggest minimising the

use of “We” to every sentence. With regard to the sample the author to specify what is meant

by adult patients, suggest giving age range.

Background:

The background of Viral load initiative well -given from WHO perspective.

“To improve ART effectiveness, the WHO recommended rapid ART initiation

within seven days of diagnosis, which has been shown to boost retention in

care and treatment outcomes (8, 11-14).UNAIDS pointed out that the

introduction of same-day ART initiation was one of the most important

cornerstones for the successful achievement of the 90-90-90 targets (15).

However, studies on the effects of rapid ART initiation have produced

inconsistent results. Some studies report benefits such as increased

retention in care (16-24), reduced morbidity and mortality (20,25-32), and

rapid and/or sustained viral suppression (17, 21-24, 29, 33-36), while

others show lower adherence (37, 38), higher loss to follow-up or no improvement in outcomes (13, 24, 39-54).”

Suggest if the authors can indicate those studies in International and

African context, by indicating the type of studies, the participants and the

findings. Presentation to be funnel type ending with where the study was

conducted, or the problem was identified. Which theory underpinned this

study, this need to be indicated.

Methods and Materials:

Well- presented, with elaboration of the setting, sampling with final selection of simple random

sampling and where the information was found being from the registers of facilities. The

sample was adult patient. and the concern is of 15 years, can 15 years old being a

teenager be regarded as an adult? This is a serious concern. The authors to give an

answer to this concern, looking also at ethical implications. Independent and dependent

variables guides were indicated. Key concepts were well-defined. Data collectors were used

and were trained, and the tool was pre-tested for validity. Data was analysed descriptively.

The Kaplan-Meier was used. Suggest that logic regression to be considered based on the

indicated variables.

Ethical considerations:

Elaboratively tabulated.

Results:

Table 1 Sociodemographic details. The age-range of from 15 years is a concern. Refer

to comments in methods and materials. Otherwise, the results were well-presented

although in the analysis of logic regression was not mentioned in data analysis section.

I have commented about that in data analysis section. Ensure consistency

Discussion

The results were discussed however, they need to be supported with other studies. Limited

information was given in this regard have noted author Chachu as cited.

Conclusion

Well- presented, with the final results and its contribution. Suggest that authors can indicate

future studies.

Technical. The authors to adhere to journal author Guidelines.

This is an interesting study which is worth to be published. Acceptance is recommended after

attendance of the indicate minor corrections

Reviewer #2: Reviewer’s Report

Manuscript Title: Viral suppression among adults living with HIV in Ethiopia during the test-and-treat era

Article Type: Research Article

General Assessment

This manuscript addresses a timely and important issue in HIV care in Ethiopia—the rate and predictors of viral suppression in the test-and-treat era. The study design is appropriate, analyses are rigorous, and the findings have clear programmatic relevance. However, the manuscript would benefit from greater clarity in its presentation, a more concise narrative in some sections, and stronger contextualization of findings within Ethiopia’s HIV program landscape. Below are my detailed comments.

Major Comments

1. Abstract

• The phrase “further improvements are needed to achieve the End HIV epidemic target of 95% suppressed viral load” is vague consider rephrasing to specify Ethiopia’s performance gap.

• The abstract is well-structured but somewhat speculative in its conclusion. The claim that higher suppression rates among advanced-disease patients “may reflect more intensive management” should either be toned down or moved to the discussion.

• Results should consistently present both percentages and absolute numbers (e.g., “290 [70.1%] of 414 clients”).

2. Introduction

• Change the Background to Introduction as this section is the summary of the whole introduction

• The introduction provides a broad overview of HIV epidemiology and treatment goals but could be more concise. There is repetition regarding UNAIDS 90-90-90 and 95-95-95 targets.

• The knowledge gap needs to be sharpened earlier. Ethiopia’s current national viral suppression rates (from DHS, EPHI, or UNAIDS reports) should be explicitly cited to establish context.

• Please clarify how this study adds value compared to recent Ethiopian studies (e.g., Belete et al. 2024; Chirnet et al. 2024; Erjino et al. 2023).

3. Methods

• The sampling procedure needs clarification. It appears stratified sampling was first used at the facility level followed by simple random sampling within facilities. Please clarify.

• Inclusion/exclusion criteria should be stated more explicitly. For example, patients without baseline viral load were excluded, but this is only later noted as a limitation.

• The measure of adherence (self-report at first appointment) is weak and prone to bias. This limitation should be acknowledged in the methods as well as in the discussion.

• Ethical clearance is mentioned, but the approval number is not provided—this should be added.

4. Results

• In Survival analysis–based studies like this one, it is very important to present and interpret both events (patients who achieved viral suppression) and censored cases (patients who did not achieve suppression during follow-up, were lost to follow-up, transferred out, or died).

• The results section is comprehensive, but the narrative repeats information already presented in tables. For clarity, the text should highlight only the most important findings.

• Attrition (loss to follow-up, deaths, transfer-outs) deserves more emphasis, given its potential impact on suppression rates.

• Interaction terms are briefly mentioned but not well explained. For instance, the reported combined effect of WHO stage and functional status is not clearly presented in the text. Nutritional status*Adherence - specified as Mild*Not good is not clear. Nutritional status is not classified as mild or moderate. You have to specify as undernourished or not.

• Rates of suppression (incidence rates vs. cumulative proportions) are sometimes difficult to follow. A clearer distinction between these metrics is recommended.

• Best practice

• You need to Present KM curves only for variables of primary interest (the study’s main predictors, significant or nearly significant ones).

• For non-significant predictors, a good compromise is:

o Mention in the methods/results that KM curves were examined for all candidate predictors, but only significant (or key clinical) ones are shown.

But in your report especially in the final model, most of the variables were not even candidate for the Multivariable Cox-regression.

5. Discussion

• The discussion contextualizes findings against regional and global studies but is sometimes speculative. The statement about advanced-disease patients achieving faster suppression due to “more intensive care” should be tempered unless supported by references.

• Comparative analysis with Ethiopia-specific studies could be expanded, highlighting differences in study setting, design, or population.

• The policy implications should be stronger and more actionable for example:

o Prioritizing same-day ART initiation through strengthened counseling and system support.

o Expanding nutritional support not only for moderate/severe malnutrition but also for mild malnutrition.

o Enhancing adherence support for patients at earlier stages of disease who may otherwise delay viral suppression.

• The limitations section is appropriate but could mention missing baseline viral load data more prominently, as this is a critical factor influencing suppression dynamics.

Minor Comments

1. Ensure consistency in reporting percentages (e.g., sometimes one decimal place, sometimes two).

2. In tables, consider reordering variables for readability (e.g., functional status and WHO stage together).

3. Improve grammar/phrasing in some areas, e.g., “clients whose viral load was determined” → “clients with available viral load measurements.”

4. Figures and tables should be self-explanatory; table captions could be expanded slightly to clarify denominators.

5. Consider condensing overly detailed background statistics (e.g., global HIV numbers) to focus more on Ethiopia.

**********

what does this mean?). If published, this will include your full peer review and any attached files.

If you choose “no”, your identity will remain anonymous but your review may still be made public.

**Do you want your identity to be public for this peer review?**  For information about this choice, including consent withdrawal, please see our Privacy Policy

Reviewer #1: No

Reviewer #2: **Yes:**  Dr. Dereje Tsegaye

**********

[NOTE: If reviewer comments were submitted as an attachment file, they will be attached to this email and accessible via the submission site. Please log into your account, locate the manuscript record, and check for the action link "View Attachments". If this link does not appear, there are no attachment files.]

While revising your submission, please upload your figure files to the Preflight Analysis and Conversion Engine (PACE) digital diagnostic tool, https://pacev2.apexcovantage.com/. PACE helps ensure that figures meet PLOS requirements. To use PACE, you must first register as a user. Registration is free. Then, login and navigate to the UPLOAD tab, where you will find detailed instructions on how to use the tool. If you encounter any issues or have any questions when using PACE, please email PLOS at figures@plos.org

Attachment Submitted filename: 20250526 comments 25-07030.pdf

Attachment Submitted filename: Comments to the authors.docx

# Author comment: Author response to Decision Letter 1

**DOI:** 10.1371/journal.pone.0362375.r002

5 Nov 2025

The detailed responses to the reviewers’ comments have been uploaded in the “Attach Files” section, as they are too extensive to be included here.

Attachment Submitted filename: Response to Reviewers.docx

# Peer review: Decision Letter 1

**DOI:** 10.1371/journal.pone.0362375.r003

**Authors:** Tebelay Dilnessa

17 Aug 2026

Dear Dr. Hebo,

Thank you for submitting your manuscript to PLOS ONE. After careful consideration, we feel that it has merit but does not fully meet PLOS ONE’s publication criteria as it currently stands. Therefore, we invite you to submit a revised version of the manuscript that addresses the points raised during the review process.

Please submit your revised manuscript by Oct 01 2026 11:59PM. If you will need more time than this to complete your revisions, please reply to this message or contact the journal office at plosone@plos.org. When you're ready to submit your revision, log on to https://www.editorialmanager.com/pone/ and select the 'Submissions Needing Revision' folder to locate your manuscript file.

- A letter that responds to each point raised by the academic editor and reviewer(s). You should upload this letter as a separate file labeled 'Response to Reviewers'.
- A marked-up copy of your manuscript that highlights changes made to the original version. You should upload this as a separate file labeled 'Revised Manuscript with Track Changes'.
- An unmarked version of your revised paper without tracked changes. You should upload this as a separate file labeled 'Manuscript'.

If you would like to make changes to your financial disclosure, please include your updated statement in your cover letter. Guidelines for resubmitting your figure files are available below the reviewer comments at the end of this letter.

If applicable, we recommend that you deposit your laboratory protocols in protocols.io to enhance the reproducibility of your results. Protocols.io assigns your protocol its own identifier (DOI) so that it can be cited independently in the future. For instructions see: https://journals.plos.org/plosone/s/submission-guidelines#loc-laboratory-protocols. Additionally, PLOS ONE offers an option for publishing peer-reviewed Lab Protocol articles, which describe protocols hosted on protocols.io. Read more information on sharing protocols at https://plos.org/protocols?utm_medium=editorial-email&utm_source=authorletters&utm_campaign=protocols.

As the corresponding author, your ORCID iD is verified in the submission system and will appear in the published article. PLOS supports the use of ORCID, and we encourage all coauthors to register for an ORCID iD and use it as well. Please encourage your coauthors to verify their ORCID iD within the submission system before final acceptance, as unverified ORCID iDs will not appear in the published article. *Only*  the individual author can complete the verification step; PLOS staff *cannot*  verify ORCID iDs on behalf of authors.

We look forward to receiving your revised manuscript.

Kind regards,

Tebelay Dilnessa, MSc

Academic Editor

PLOS One

Journal Requirements:

If the reviewer comments include a recommendation to cite specific previously published works, please review and evaluate these publications to determine whether they are relevant and should be cited. There is no requirement to cite these works unless the editor has indicated otherwise.

Please review your reference list to ensure that it is complete and correct. If you have cited papers that have been retracted, please include the rationale for doing so in the manuscript text, or remove these references and replace them with relevant current references. Any changes to the reference list should be mentioned in the rebuttal letter that accompanies your revised manuscript. If you need to cite a retracted article, indicate the article’s retracted status in the References list and also include a citation and full reference for the retraction notice.

Additional Editor Comments:

- The comments were addressed well, but still it requires a careful revision.
- In table 3, all variables should have values. For example, rapid ART initiation, Yes=A; No=B
- Figures 3-5 were well cited. But the concept for each respective figure was too short.
- Line 112: **Materials and Methods**
- Line 113: **Study area and setting**
- Add the p-value to the description part
- The list of abbreviations (no need for two columns) and the declaration section should be above the list of references.
- The reference list should be written based on the standard format.
- Upload figures in a separate file as TIF/or TIFF version without their captions, captions remain within the main manuscript where cited and at list of ‘supporting’ information.
- What is your basis for citing the ‘supporting information’ in the ‘Data analysis’ section?

[Note: HTML markup is below. Please do not edit.]

Reviewers' comments:

Reviewer's Responses to Questions

**Comments to the Author**

Reviewer #3: (No Response)

**********

2. Is the manuscript technically sound, and do the data support the conclusions?

Reviewer #3: Partly

**********

3. Has the statistical analysis been performed appropriately and rigorously?

Reviewer #3: Yes

**********

4. Have the authors made all data underlying the findings in their manuscript fully available?

The PLOS Data policy

Reviewer #3: Yes

**********

5. Is the manuscript presented in an intelligible fashion and written in standard English?

Reviewer #3: Yes

**********

Reviewer #3: 1. The introduction of the abstract should be 1–2 sentences that state the public health problem, clinical gap, or knowledge gap your study addresses. Stating the systematic review in the introduction in not adressing the gap. please revise

2. Please clearly indicate what time point is the suppression rate is measured in objective and in the results of the abstract. This is also valid for the main article where follow up time is referred as time point.

3. In the methods of abstract it says the patients were followed up for 2 years. But in the text it says patients starting ART until Dec 2021 were included and followed up until Dec 22. So that means there is less that 2 years of follow up for some.

4. The anderson methods is described in the introduction. It should be in methods.

5. Could you clarify if the censored cases includes those who did not achieve the viral suppression? IF yes please revise the definition.

6. The result section of the viral suppression rate is little confusing. For better visualisation, I suggest using a flowchart so we can easily follow up the numbers. And present clearly the suppression in terms of time point.

7. The discussion indeed is more rich but could be better written. The section where the comparisation is made with others studies that have same results could be more summarised and easy to read. If there is specific characteristic of the study it could be emphasise. Again what additional knowledge this study is brought should be emphasised.

Best Regards

**********

what does this mean?). If published, this will include your full peer review and any attached files.

If you choose “no”, your identity will remain anonymous but your review may still be made public.

**Do you want your identity to be public for this peer review?**  For information about this choice, including consent withdrawal, please see our Privacy Policy

Reviewer #3: No

**********

[NOTE: If reviewer comments were submitted as an attachment file, they will be attached to this email and accessible via the submission site. Please log into your account, locate the manuscript record, and check for the action link "View Attachments". If this link does not appear, there are no attachment files.]

To ensure your figures meet our technical requirements, please review our figure guidelines: https://journals.plos.org/plosone/s/figures

You may also use PLOS’s free figure tool, NAAS, to help you prepare publication quality figures: https://journals.plos.org/plosone/s/figures#loc-tools-for-figure-preparation.

NAAS will assess whether your figures meet our technical requirements by comparing each figure against our figure specifications.

# Author comment: Author response to Decision Letter 2

**DOI:** 10.1371/journal.pone.0362375.r004

7 Sep 2026

We have uploaded responses to the reviewer and editor. We could not paste here the contents of response letter as it contains diagram.

Attachment Submitted filename: Response_to_Reviewers_auresp_2.docx

# Peer review: Decision Letter 2

**DOI:** 10.1371/journal.pone.0362375.r005

**Authors:** Tebelay Dilnessa

26 Oct 2026

Viral suppression among adolescents and adults living with human immunodeficiency virus in Ethiopia during the test-and-treat era

PONE-D-25-07030R2

Dear Dr. Hebo,

We’re pleased to inform you that your manuscript has been judged scientifically suitable for publication and will be formally accepted for publication once it meets all outstanding technical requirements.

Within one week, you’ll receive an e-mail detailing the required amendments. When these have been addressed, you’ll receive a formal acceptance letter and your manuscript will be scheduled for publication.

An invoice will be generated when your article is formally accepted. Please note, if your institution has a publishing partnership with PLOS and your article meets the relevant criteria, all or part of your publication costs will be covered. Please make sure your user information is up-to-date by logging into Editorial Manager at Editorial Manager® and clicking the ‘Update My Information' link at the top of the page. For questions related to billing, please contact billing support.

If your institution or institutions have a press office, please notify them about your upcoming paper to help maximize its impact. If they’ll be preparing press materials, please inform our press team as soon as possible -- no later than 48 hours after receiving the formal acceptance. Your manuscript will remain under strict press embargo until 2 pm Eastern Time on the date of publication. For more information, please contact onepress@plos.org.

Kind regards,

Tebelay Dilnessa, MSc

Academic Editor

PLOS One

Additional Editor Comments (optional):

Reviewers' comments:

Reviewer's Responses to Questions

**Comments to the Author**

Reviewer #3: All comments have been addressed

**********

2. Is the manuscript technically sound, and do the data support the conclusions?

Reviewer #3: Yes

**********

3. Has the statistical analysis been performed appropriately and rigorously?

Reviewer #3: Yes

**********

4. Have the authors made all data underlying the findings in their manuscript fully available?

The PLOS Data policy

Reviewer #3: Yes

**********

5. Is the manuscript presented in an intelligible fashion and written in standard English?

Reviewer #3: Yes

**********

Reviewer #3: (No Response)

**********

what does this mean?). If published, this will include your full peer review and any attached files.

If you choose “no”, your identity will remain anonymous but your review may still be made public.

**Do you want your identity to be public for this peer review?**  For information about this choice, including consent withdrawal, please see our Privacy Policy

Reviewer #3: **Yes:**  Bindiya Meggi

**********

# Editor report: Acceptance letter

**DOI:** 10.1371/journal.pone.0362375.r006

**Authors:** Tebelay Dilnessa

PONE-D-25-07030R2

PLOS One

Dear Dr. Hebo,

I'm pleased to inform you that your manuscript has been deemed suitable for publication in PLOS One. Congratulations! Your manuscript is now being handed over to our production team.

At this stage, our production department will prepare your paper for publication. This includes ensuring the following:

* All references, tables, and figures are properly cited

* All relevant supporting information is included in the manuscript submission,

* There are no issues that prevent the paper from being properly typeset

You will receive further instructions from the production team, including instructions on how to review your proof when it is ready. Please keep in mind that we are working through a large volume of accepted articles, so please give us a few days to review your paper and let you know the next and final steps.

Lastly, if your institution or institutions have a press office, please let them know about your upcoming paper now to help maximize its impact. If they'll be preparing press materials, please inform our press team within the next 48 hours. Your manuscript will remain under strict press embargo until 2 pm Eastern Time on the date of publication. For more information, please contact onepress@plos.org.

You will receive an invoice from PLOS for your publication fee after your manuscript has reached the completed accept phase. If you receive an email requesting payment before acceptance or for any other service, this may be a phishing scheme. Learn how to identify phishing emails and protect your accounts at https://explore.plos.org/phishing.

If we can help with anything else, please email us at customercare@plos.org.

Thank you for submitting your work to PLOS One and supporting open access.

Kind regards,

PLOS One Editorial Office Staff

on behalf of

Dr. Tebelay Dilnessa

Academic Editor

PLOS One
